# Changes in Epidemiology of Candidemia in the United States With a Focus on *Candida auris*

**DOI:** 10.1093/cid/ciag312

**Published:** 2026-05-11

**Authors:** María Alejandra Pérez, Khush A Patel, Laila Rasmy, Hisato Yoshida, Cesar A Arias, Masayuki Nigo, Max W Adelman

**Affiliations:** Center for Infectious Diseases, Houston Methodist Research Institute, Houston, Texas, USA; Department of Surgery, Houston Methodist Hospital, Houston, Texas, USA; McWilliams School of Biomedical Informatics, UTHealth at Houston, Houston, Texas, USA; Center for Infectious Diseases, Houston Methodist Research Institute, Houston, Texas, USA; Department of Dentistry and Oral Surgery, Unit of Sensory and Locomotor Medicine, Division of Medicine, Faculty of Medical Sciences, University of Fukui, Fukui, Japan; Center for Infectious Diseases, Houston Methodist Research Institute, Houston, Texas, USA; Division of Infectious Diseases, Department of Medicine, Houston Methodist Hospital, Houston, Texas, USA; Department of Medicine, Weill Cornell Medical College, NewYork, New York, USA; Center for Infectious Diseases, Houston Methodist Research Institute, Houston, Texas, USA; Division of Infectious Diseases, Department of Medicine, Houston Methodist Hospital, Houston, Texas, USA; Department of Medicine, Weill Cornell Medical College, NewYork, New York, USA; Center for Infectious Diseases, Houston Methodist Research Institute, Houston, Texas, USA; Division of Infectious Diseases, Department of Medicine, Houston Methodist Hospital, Houston, Texas, USA; Department of Medicine, Weill Cornell Medical College, NewYork, New York, USA; Division of Pulmonary, Critical Care and Sleep Medicine, Department of Medicine, Houston Methodist Hospital, Houston, Texas, USA

**Keywords:** *Candida*, *Candida auris*, candidemia, epidemiology, drug resistance

## Abstract

**Background:**

Candidemia is a common complication in hospitalized and critically ill patients. Nationwide US data on candidemia risk factors, species, antifungal resistance, and outcomes remain limited. Our objective was to describe candidemia trends from 2015 to 2024 and identify risk factors for *Candida auris* candidemia.

**Methods:**

Retrospective study using the Epic Cosmos database. We analyzed trends in candidemia risk factors and outcomes. Thirty-day mortality was evaluated using multivariable logistic regression adjusted for age, Elixhauser comorbidity index, COVID-19–related hospitalization, ICU-acquired candidemia, and calendar year. We compared characteristics of patients with *C. auris* vs. non-*C. auris* candidemia.

**Results:**

A total of 29 454 candidemia cases were identified among 27 926 unique patients; candidemia incidence increased over time (*P* = .02). Overall 30-day mortality was 31.5% and increased significantly over the study period (from 24.7% to 31.8%; *P* = .02). There were similar trends in in-hospital mortality (*P* = .01) and intensive care unit–acquired candidemia (*P* = .03). The proportion of cases involving *C. albicans* decreased (*P* = .05) while *C. auris* cases increased (*P* = .008). Patients with *C. auris* candidemia were more likely to be Black, have renal failure, and have a higher social vulnerability index (*P* < .001 for all comparisons). Fluconazole resistance increased in *C. parapsilosis* (from 2.5% to 10.8%, *P* = .02) and *C. tropicalis* (from 6.2% to 18.1%, *P* = .03).

**Conclusions:**

Candidemia-associated 30-day mortality increased over the past decade, aligning with a trend toward older, more acutely ill patients developing candidemia. *Candida auris* candidemia was associated with Black race, higher social vulnerability, and renal failure. Overall, our findings highlight concerning nationwide US candidemia trends.

## Teaser

In this nationwide study, candidemia was associated with increasing 30-day mortality over the past decade. There were trends toward more elderly and critically ill patients developing candidemia. Patients with *C. auris* candidemia had higher social vulnerability and renal failure.

*Candida* spp. cause an estimated 23 000 bloodstream infections yearly in the United States [1], and *Candida* bloodstream infections (candidemia) are associated with 25%–40% mortality [1–3]. This high mortality is in part due to the propensity for candidemia to affect medically vulnerable and chronically ill patients [1, 2, 4]. Nationwide candidemia data are crucial to understand trends in risk factors, species, antifungal resistance, and outcomes.

The incidence of candidemia increased during the COVID-19 pandemic, likely due to increased acuity of illness, length of stay, and use of invasive medical devices, all known risk factors for candidemia [2, 5, 6]. During COVID-19, there was also increased candidemia-related mortality [2, 7], which may be related both to high candidemia incidence among patients with COVID-19 and more elderly patients with comorbidities developing candidemia. It is unknown if the COVID-era changes in risk factors and mortality have persisted after the pandemic.

Along with changing demographics and outcomes, several studies have reported changes in causative species among patients with candidemia [4, 8]. *Candida albicans* has long been the most common species [2, 4]. However, there has been an increase in the prevalence of *Nakaseomyces glabratus* (formerly *C. glabrata*) [2, 9], and the commonly multidrug-resistant *C. auris* has continued to emerge globally [10, 11]. Lastly, while species such as *Pichia kudriavzevii* (formerly *C. krusei*) and *C. auris* have intrinsic antifungal resistance, there have been reports of increasing resistance among generally drug-susceptible species such as *C. parapsilosis* [12].

While these reports have raised concern, there has been no nationwide study addressing changes in candidemia epidemiology after the height of the COVID-19 pandemic, and prior studies have included only small numbers of *C. auris* cases [2]. To inform efforts at candidemia prevention and treatment, we used the Epic Cosmos database, which includes electronic health data for 300 million US patients [13], to analyze trends in candidemia epidemiology between 2015 and 2024.

## METHODS

### Study Design

We conducted a retrospective cohort study using Epic Cosmos [13]. The Houston Methodist Institutional Review Board determined that the study was exempt. We included patients with a blood culture from 2015 to 2024 positive for an organism of the genus *Candida* or newly designated genera (*Nakaseomyces*, *Pichia*, *Meyerozyma*, *Clavispora*, *Diutina*, *Candidozyma*, *Debaryomyces*, and *Kluyveromyces*) [14]. Given that Epic Cosmos updates continuously, we fixed the dataset on 23 August 2025. We extracted demographics, hospital and intensive care unit (ICU) encounters, comorbidities, and a history of COVID-19 infection. The primary outcomes were 30-day mortality, in-hospital mortality, and ICU-acquired candidemia.

### Operational Definitions

Following the Centers for Disease Control and Prevention (CDC) definitions [2], we considered any positive blood culture within 30 days of the first positive blood culture to be part of the same candidemia case, and any positive blood culture after 30 days was considered a new case. Multiple *Candida* species isolated within the 30-day window, if present, were attributed to the same case. The index date was defined as the collection date of the first positive blood culture.

Patients with more than one case were classified as having recurrent candidemia [15].

Cases were considered community onset if the index blood culture was collected outside of a recorded hospital admission or on days 1–2 of hospital admission, and healthcare onset if collected ≥3 days [2]. For each case, hospitalization within 90 days of the index date was assessed [2], and discharge destination after the most recent hospitalization was used to identify nursing home or long-term care hospital exposure prior to the index admission.

ICU stays were considered if lasting >1 calendar day and categorized as (1) ICU stay during hospitalization and (2) ICU stay during the 13 days after the index date (per CDC definitions) [2]. Cases were considered ICU-acquired if the index date occurred on day 3 or later of ICU admission or less than 2 days after ICU discharge [16].

### Statistical Analysis

Demographics, comorbidities, and the Elixhauser comorbidity index (ECI) [17] were assessed at the patient level using the index date of the first case. Outcomes, including 30-day mortality, in-hospital mortality, and ICU requirement, as well as COVID-19–related hospitalization and infection onset, were assessed per case as per CDC reporting [2]. The Mann–Kendall test was used to determine temporal trends. The proportion of species was calculated by dividing the total number of cases caused by each species each year by the total number of candidemia cases that year.

We fit a multivariable logistic regression model for 30-day mortality. Covariates included age, ECI, COVID-19–related hospitalization, ICU-acquired candidemia, and calendar year. Health-system fixed effects were included to control for baseline differences between systems, and two-way cluster-robust standard errors at the patient and health-system levels were used to account for multiple episodes per patient and correlation among patients treated within the same system [18].

For antifungal susceptibility analysis, resistance rates for each antifungal were calculated using the total number of isolates tested against that antifungal as the denominator. Resistance rates were estimated for species-antifungal combinations with defined Clinical and Laboratory Standards Institute (CLSI) or CDC breakpoints [19, 20]. We also analyzed resistance to any echinocandin (anidulafungin, caspofungin, or micafungin) and multidrug resistance (resistance to ≥1 echinocandin plus fluconazole) [2]. Resistance rates were also analyzed per CDC-defined geographic region [21].

### Subgroup Analyses

We compared patients with *C. auris* candidemia to those with non-*C. auris* candidemia. For the *C. auris* cohort, patients were included if they had a case attributed exclusively *to C. auris*; analyses were restricted to those *C. auris* cases. We then identified the health systems that reported these cases and drew a comparison group from the same systems, limited to 2019–2024 to match the *C. auris* case period, comprising patients with cases due to other *Candida* species and no history of *C. auris*. To compare groups, categorical variables were tested using the Chi-square test. For numerical variables, normality was assessed with the Lilliefors test. If both groups were normally distributed, we used a t-test; otherwise, a Wilcoxon rank-sum test. All analyses were performed in R (version 4.1.1) using the dplyr, stats, cobalt, fixest, and lme4 packages [22]. Methods for sensitivity analyses and additional information on variable definitions and handling of missing data are described in the Methods of the supplementary material.

## RESULTS

### Patients and Demographics

We identified 29 454 candidemia cases from 2015 to 2024 among 27 926 unique patients across 141 health systems. Patients per state are shown in the Supplementary Figure 1; these counts reflect each state's contribution to the cohort and should not be interpreted as reflecting their candidemia burden. Overall demographics are shown in Table 1. Among 27 926 unique patients, the mean age was 58.9 years (SD 19.5), and 15 523 (55.6%) were male. A total of 19 637 patients (70.3%) were White, and 5827 (20.9%) were Black; 2159 (7.9%) were Hispanic or Latino. Mean age increased from 2015 to 2024 (54.4 years ± SD 21.4 to 61.4 ± 19.0, *P* = .0007), as did the proportion of Black patients (17.1% to 22.9%, *P* = .004) and Hispanic or Latino patients (3.6% to 9.1%, *P* = .004). There was no significant change in gender distribution over time; these demographic trends are shown in Supplementary Figures 2–5. Mean ECI was 13.1 (SD 14.3) and did not change over the study period (*P* = .72). The most common comorbidities across all years were anemia (40.5%, 11 300/27 926), uncomplicated diabetes (38.9%, 10 835/27 926), and uncomplicated hypertension (33.2%, 9345/27 926). Renal failure, both moderate (19.3%, 5382/27 926) and severe (10.9%, 3069/27 926), was also common.

**Table 1.** Demographics, Risk Factors, and Outcomes Among N = 27 926 Patients With Candidemia Identified Across 141 US Health Systems, 2015–2024

Variable	Value
Age, years, mean (SD)	58.9 (19.5)
Age ≥18 y	26 876 (96.2%)
Sex, N (%)	
Female	12 386 (44.3%)
Male	15 523 (55.6%)
Unknown	17 (0.1%)
Race, N (%)^a^	
American Indian/Alaska Native	431 (1.5%)
Asian	633 (2.3%)
Black/African American	5827 (20.9%)
Native Hawaiian/Pacific Islander	102 (0.4%)
Other	776 (2.8%)
White	19 637 (70.3%)
Missing	520 (1.9%)
Ethnicity	
Not Hispanic/Latino	24 528 (87.8%)
Hispanic/Latino	2195 (7.9%)
Unspecified	1203 (4.3%)
SVI, overall percentile, median (IQR)^b^	0.69 (0.42–0.88)
Onset^c^	
Community onset	13 198 (44.8%)
Healthcare onset	16 256 (55.2%)
Recent hospitalization (≤90 d)^c^	11 682 (39.7%)
Most recent discharge disposition^d^	
Nursing home	2286 (19.6%)
Long-term care hospital	324 (2.8%)
ICU-acquired^c^	6813 (23.1%)
ICU stay during hospitalization^c^	15 292 (51.9%)
ICU stay during the 13 d after the index date^c^	11 862 (40.3%)
COVID-19–related hospitalization^c^	1916 (6.5%)
Recurrence	1110 (3.9%)
Mortality^c^	
In-hospital	7784 (26.4%)
30-day	9282 (31.5%)
ECI, mean (SD)^e^	13.1 (14.3%)
Comorbidities^e^	
Anemias	11 300 (40.5%)
Diabetes, uncomplicated	10 835 (38.9%)
Hypertension, uncomplicated	9345 (33.2%)
Coagulopathy	7610 (27.2%)
Weight loss	7406 (26.5%)
Renal failure, moderate	5382 (19.3%)
Solid tumor without metastasis, malignant	5115 (18.3%)
Peripheral vascular disease	4672 (16.7%)
Chronic pulmonary disease	3911 (14.0%)
Heart failure	3810 (13.6%)
Liver disease, mild	3716 (13.3%)
Valvular disease	3701 (13.2%)
Hypothyroidism	3353 (12.0%)
Renal failure, severe	3069 (10.9%)
Diabetes, complicated	2914 (10.4%)
Obesity	2745 (9.8%)
Cerebrovascular disease	2295 (8.2%)
Chronic blood loss anemia (iron deficiency)	1947 (6.9%)
Metastatic cancer	1417 (5.0%)
Peptic ulcer disease	1390 (4.9%)
Hypertension, complicated	1368 (4.9%)
Pulmonary circulation disease	1336 (4.8%)
Depression	1068 (3.8%)
Other thyroid disorders	1050 (3.8%)
Liver disease, moderate to severe	1006 (3.6%)
Other neurological disorders	786 (2.8%)
Autoimmune conditions	818 (2.9%)
Leukemia	595 (2.1%)
Paralysis	603 (2.1%)
Psychosis	571 (2.0%)
Neurological disorders affecting movement	562 (2.0%)
Seizures and epilepsy	410 (1.5%)
Solid organ transplant	404 (1.4%)
Dementia	365 (1.3%)
Lymphoma	322 (1.1%)
Drug abuse	254 (0.9%)
Acquired immune deficiency syndrome	179 (0.6%)
Solid tumor without metastasis, in situ	180 (0.6%)
Alcohol abuse	82 (0.3%)

Abbreviations: ICU, intensive care unit; IQR, interquartile range (25th–75th percentile); SD, standard deviation.

^a^Race in Epic Cosmos is self-reported according to the CDC categories [23].

^b^The SVI values reflect the CDC and Agency for Toxic Substances and Disease Registry (ATSDR) overall SVI percentile, which ranges from 0 to 1. Higher values indicate greater vulnerability [24]. A total of 808 patients did not have information for this variable; the median and IQR were calculated among 27 118 patients.

^c^Percentages in these categories are based on the total number of cases (N = 29 454). Other percentages are calculated based on the total number of patients (N = 27 926) as per the CDC criteria [2].

^d^Percentages for the most recent discharge disposition are based on the total of cases with previous hospitalizations within 90 days of the index date (N = 11 682).

^e^Comorbidity data were unavailable for 437 patients. Percentages are calculated based on the total number of patients (N = 27 926). Comorbidities were defined using the ECI from the International Classification of Diseases-10-Clinical Modification (ICD-10-CM) Refined Software [17]. The ECI score was calculated for each patient's first case of candidemia.

Overall candidemia incidence was 50.5 new cases per 100 000 hospital admissions, and incidence increased over time (*P* = .02) (Supplementary Figure 6). Most patients (26 816, 96.0%) developed one case; a total of 1110 patients experienced recurrence. Among 66.5% (738/1110) of these patients, the *Candida* species identified in the first episode was present in all subsequent episodes. The number of cases per patient and the species distribution among recurrent cases are presented in Supplementary Tables 1 and 2. The number of cases identified per year increased (Supplementary Table 3), in part because the number of contributing health systems increased.

### Infection Onset and Mortality

A total of 44.8% of cases (13 198/29 454) were community onset, and 55.2% (16 256/29 454) were healthcare onset (Table 1). Additionally, 39.7% of cases (11 682/29 454) had a hospitalization within 90 days of the index date; of those, 19.6% (2286/11 682) were discharged from the prior hospitalization to a nursing home and 2.8% (324/11 682) to a long-term care hospital. The proportion of healthcare onset cases peaked in 2021 (59.2%, 2211/3734) (Supplementary Figure 7). After the pandemic, healthcare onset cases decreased but remained higher than prepandemic levels, although there was no significant trend over the study period (*P* = .07).

Overall, 23.1% of cases (6813/29 454) were ICU-acquired (Table 1), and the proportion of ICU-acquired cases increased from 2015 (16.9% [173/1024]) and peaked in 2020 (28.3% [848/2998]) (Supplementary Figure 8). While there was a decline after the height of the COVID-19 pandemic, the proportion of ICU-acquired cases remained above prepandemic levels (*P* = .03 for overall trend from 2015 to 2024). There were similar trends in the proportion of patients who required any ICU stay during hospitalization (*P* = .05) or during the 13 days after the index date (*P* = .05) (Supplementary Figures 9 and 10).

Overall, 30-day mortality was 31.5% (9282/29 454), and unadjusted mortality increased significantly from 24.7% (253/1024) in 2015 to 31.8% (1609/5063) in 2024 (*P* = .02) (Figure 1). While mortality began to rise before the pandemic, the increase was more pronounced during COVID-19: 30-day mortality increased from 27.9% (722/2585) in 2019 to 35.6% (1068/2998) in 2020 before peaking at 37.6% (1404/3734) in 2021. Although 30-day mortality decreased to 31.1% (1424/4574) in 2023, it remained higher than prepandemic levels in 2023 and 2024. Similarly, the adjusted odds ratio for mortality (aOR) peaked in 2021 at 1.34 (95% CI, 1.16–1.55) and decreased to 1.19 (95% CI, 1.03–1.38) in 2024 (Figure 2). Similar trends in in-hospital mortality were also identified (Supplementary Figure 11).

**Figure 1.**
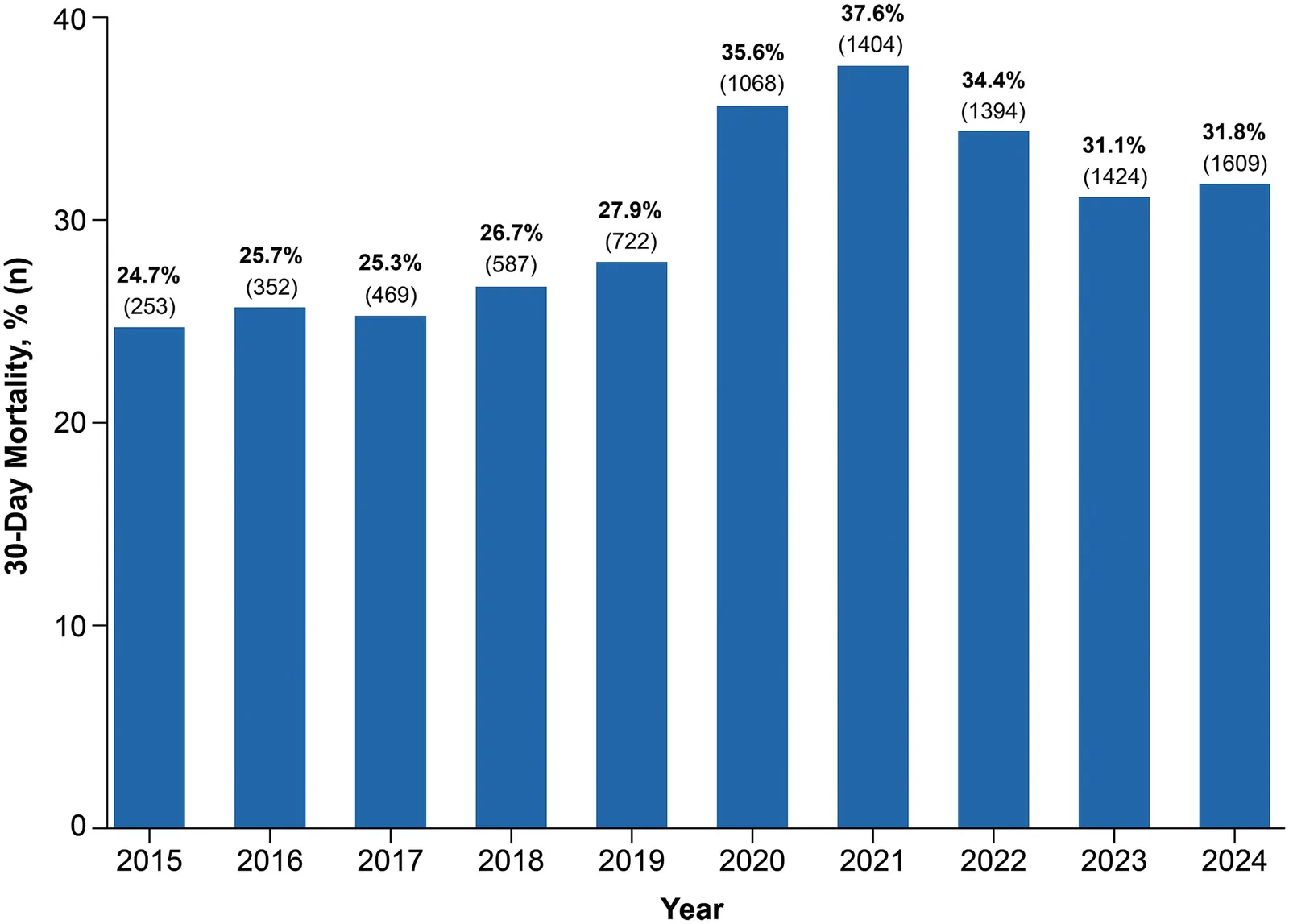
Thirty-day mortality after candidemia onset by year, 2015–2024. Legend: unadjusted mortality increased over the study period (*P* = .02). Abbreviation: n, number of cases.

**Figure 2.**
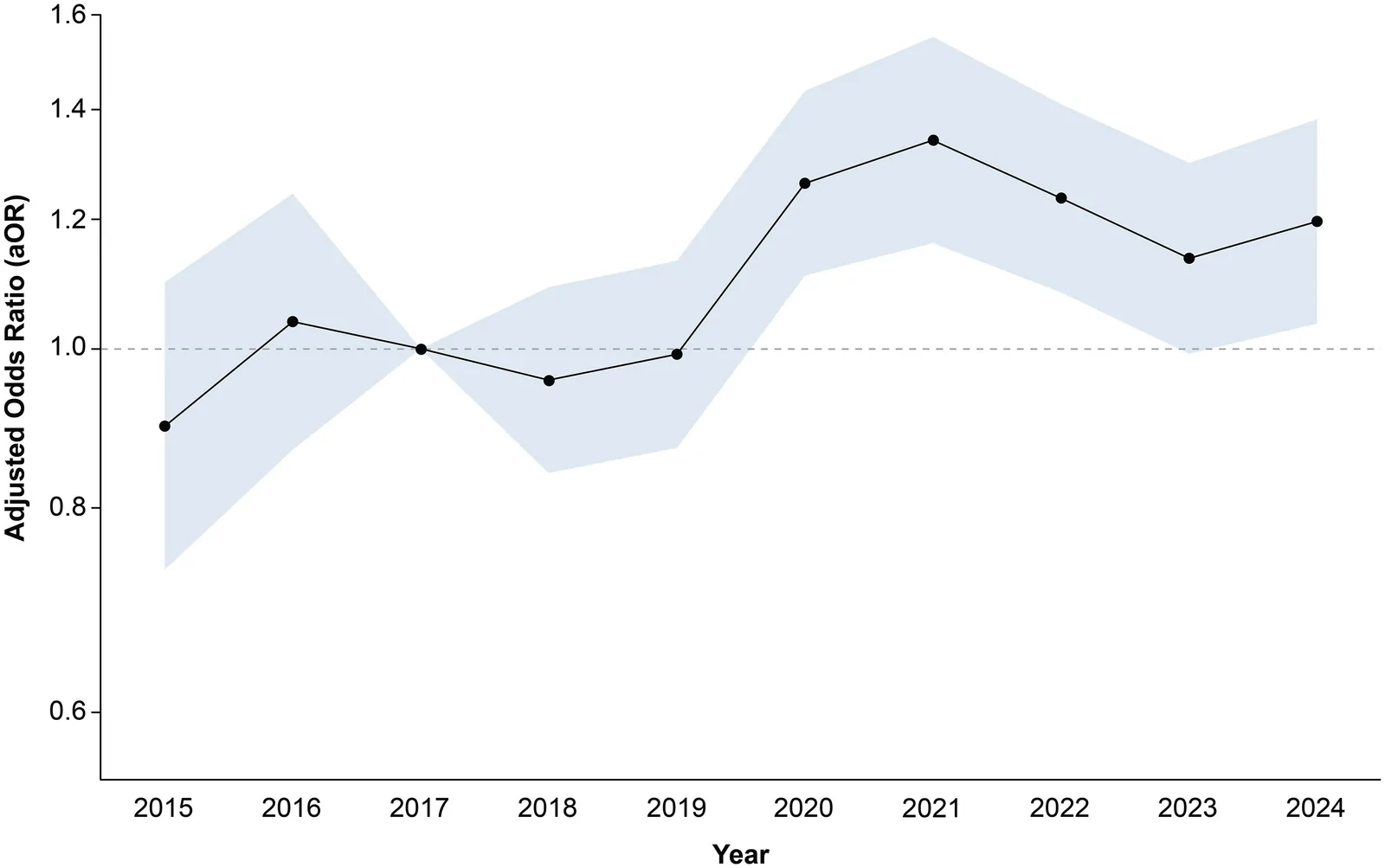
Adjusted odds of 30-day mortality after candidemia onset by year, 2015–2024. Legend: aOR for 30-day mortality was estimated using a multivariable regression model including age and ECI at each episode, COVID-19–related hospitalization, ICU-acquired candidemia, and calendar year. Health-system fixed effects were included to account for baseline differences across systems. Two-way cluster-robust standard errors at the level of patient and health system were used to account for multiple episodes per patient and within-system correlation. The shaded area indicates the 95% confidence intervals. 2017 was selected as the reference category because it matched the median adjusted mortality probability during the prepandemic era, and all aORs are presented relative to that year. Abbreviation: n, number of cases.

### Sensitivity Analysis

After restricting the population to health systems that met data quality standards (see Supplementary Methods), 13 health systems were included. The sensitivity cohort included 4061 cases among 3821 patients; cohort characteristics are shown in Supplementary Table 4; candidemia incidence, trends in demographics, risk factors, outcomes, and species distribution are shown in Supplementary Figures 12–23. Covariate balance between the main and the sensitivity cohort and concordance of temporal trends between cohorts are shown in Supplementary Tables 5–7. Adjusted odds ratios for 30-day mortality in the sensitivity cohort are presented in Supplementary Figures 24 and 25.

### Species and Antifungal Susceptibility

The most common species was *C. albicans*, identified in 40.0% (11 782/29 454) of cases. The proportion of cases involving *C. albicans* decreased from 41.7% (427/1024) in 2015 to 36.4% (1844/5063) in 2024 (*P* = .05) (Figure 3). Meanwhile, the proportion of *C. auris* cases significantly increased from 2019 to 2024 (*P* = .008). Indeed, the first *C. auris* case was reported in 2019, and by 2024, 4.7% of cases (237/5063) involved *C. auris*.

**Figure 3.**
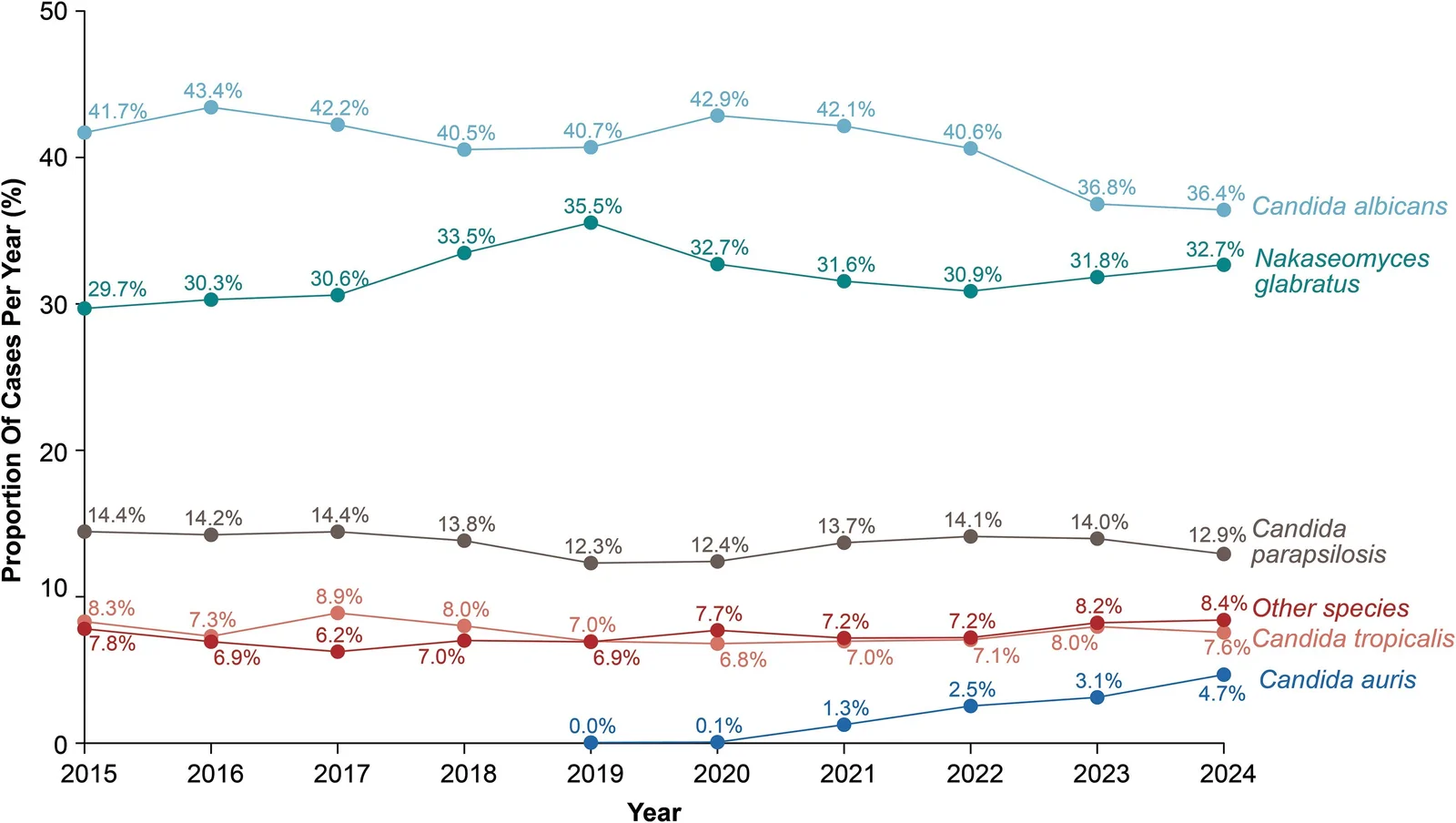
Proportion of *Candida* species cases among all candidemia cases, 2015–2024. Legend: the proportion of cases of *C. albicans* decreased over the study period (*P* = .05 by Mann–Kendall test), and the proportion of *C. auris* increased from 2019 to 2024 (*P* = .008).

After *C. albicans*, *N. glabratus* was the second most common species (32.1%, 9462/29 454), followed by *C. parapsilosis* (13.5%, 3981/29 454), *C. tropicalis* (7.5%, 2203/29 454), and *C. auris* (1.8%, 533/29 454) (Supplementary Table 1). Thirty-day mortality was highest for *C. albicans* (34.0% [4007/11 782 cases]), followed by *C. tropicalis* (33.7% [743/2203]), *N. glabratus* (32.4% [3064/9462]), *C. auris* (29.8% [159/533]), and *C. parapsilosis* (20.1% [799/3981]) (Supplementary Figure 26).

Changes in antifungal resistance by species are shown in Supplementary Tables 8–13. Resistance to fluconazole remained low for *C. albicans* (2.2% in 2024, 34/1509 isolates) and decreased over the study period in *N. glabratus* from 15.6% (33/211) to 9.6% (113/1179) (*P* = .007). Conversely, fluconazole resistance rose for both *C. parapsilosis* (from 2.5% [3/118] to 10.8% [62/574], *P* = .02) and *C. tropicalis* (from 6.2% [4/64] to 18.1% [58/321], *P* = .03). By 2024 all *C. auris* isolates (58/58) were fluconazole resistant. Echinocandin resistance remained uncommon among *C. auris* (9.1% [4/44] for micafungin, 0% [0/29] for each of anidulafungin and caspofungin). Resistance by CDC-defined geographic region is shown in Supplementary Figures 27 and 28.

### *Candida auris*-specific Risk Factors and Outcomes

Given the increase in *C. auris* cases, we compared patients with *C. auris* candidemia to patients with candidemia due to other species. We compared 457 patients with *C. auris* candidemia (514 cases) to 9920 patients with non-*C. auris* candidemia (10 386 cases) (Table 2, Supplementary Figure 29). Overall, *C. auris* was reported at 39 health systems (Supplementary Figure 30). In 2024, when 38 health systems reported cases, the median new cases per health system was 2 (range 0–24).

**Table 2.** Demographics, Risk Factors, and Outcomes Comparing Patients With Candidemia Due to *C. auris* Versus Candidemia Due to *Candida* Species Other Than *C*. *auris*

Variable	*C. auris* candidemia (N = 457)	Non-*C. auris* candidemia (N = 9920)	*P* value
Age, years, mean (SD)	62.2 (14.7)	60 (18.4)	.16
Age ≥18 y	457(100%)	9675 (97.5%)	<.001
Sex, N (%)			
Female	202 (44.2%)	4368 (44.0%)	.98
Male	255 (55.8%)	5537 (55.8%)	1.0
Unknown	0 (0%)	15 (.15%)	.84
Race, N (%)^a^			
American Indian/Alaska Native	4 (0.9%)	120 (1.2%)	.67
Asian	13 (2.8%)	240 (2.4%)	.67
Black/African American	211 (46.2%)	2235 (22.5%)	<.001
Native Hawaiian/Pacific Islander	4 (0.8%)	25 (0.2%)	.04
Other	11 (2.4%)	296 (2.9%)	.57
White	206 (45.1%)	6765 (68.2%)	<.001
Missing	8 (1.7%)	239 (2.4)	.45
Ethnicity			
Not Hispanic/Latino	392 (85.8%)	8562 (86.3%)	.79
Hispanic/Latino	38 (8.3%)	859 (8.6%)	.86
Unspecified	27 (5.9%)	499 (5.0%)	.46
SVI, overall percentile, median (IQR)^b^	0.78 (0.55–0.91)	0.71 (0.46–0.88)	<.001
Onset^c^			.03
Community onset	255 (49.6%)	4640 (44.7%)	
Healthcare onset	259 (50.4%)	5746 (55.3%)	
Recent hospitalization (≤90 d)^c^	282 (54.9%)	4120 (39.7%)	<.001
Most recent discharge disposition^d^			
Nursing home	118 (41.8%)	806 (19.6%)	<.001
Long-term care hospital	35 (12.4%)	90 (2.2%)	<.001
ICU-acquired^c^	94 (18.3%)	2435 (23.4%)	.008
ICU stay during hospitalization^c^	305 (59.3%)	5375 (51.7%)	<.001
ICU stay during the 13 d after the index date^c^	205 (39.8%)	4169 (40.1%)	.94
COVID-19–related hospitalization^c^	19 (3.7%)	791 (7.6%)	.001
Recurrence	62 (13.6%)	394 (3.9%)	<.001
Mortality^c^			
In-hospital	140 (27.2%)	2832 (27.3%)	1.0
30-day	152 (29.6%)	3379 (32.5%)	.17
ECI, mean (SD)^e^	15.9 (15.6)	13.2 (14.2)	<.001
Comorbidities^e^			
Anemias	228 (49.9%)	3885 (39.2%)	<.001
Diabetes, uncomplicated	220 (48.1%)	4026 (40.6%)	.002
Hypertension, uncomplicated	165 (36.1%)	3077 (31.0%)	.002
Coagulopathy	125 (27.3%)	2669 (26.9%)	.87
Weight loss	149 (32.6%)	2729 (27.5%)	.02
Renal failure, moderate	111 (24.3%)	1783 (18.0%)	<.001
Solid tumor without metastasis, malignant	56 (12.2%)	1843 (18.6%)	<.001
Peripheral vascular disease	86 (18.8%)	1599 (16.1%)	.14
Chronic pulmonary disease	63 (13.8%)	1344 (13.5%)	.94
Heart failure	109 (23.8%)	1405 (14.1%)	<.001
Liver disease, mild	42 (9.2%)	1321 (13.3%)	.013
Valvular disease	68 (14.9%)	1289 (13.0%)	.27
Hypothyroidism	61 (13.3%)	1103 (11.1%)	.16
Renal failure, severe	140 (30.6%)	1130 (11.4%)	<.001
Diabetes, complicated	60 (13.1%)	967 (9.7%)	.02
Obesity	80 (17.5%)	1102 (11.1%)	<.001
Cerebrovascular disease	53 (11.6%)	826 (8.3%)	.01
Chronic blood loss anemia (iron deficiency)	39 (8.5%)	707 (7.1%)	.29
Metastatic cancer	4 (0.9%)	511 (5.1%)	<.001
Peptic ulcer disease	16 (3.5%)	456 (4.6%)	.32
Hypertension, complicated	18 (3.9%)	373 (3.8%)	.94
Pulmonary circulation disease	39 (8.5%)	472 (4.8%)	<.001
Depression	11 (2.4%)	386 (3.9%)	.13
Other thyroid disorders	24 (5.2%)	387 (3.9%)	.18
Liver disease, moderate to severe	18 (3.9%)	373 (3.8%)	.94
Other neurological disorders	18 (3.9%)	248 (2.5%)	.08
Autoimmune conditions	10 (2.2%)	297 (3.0%)	.39
Leukemia	3 (0.6%)	192 (1.9%)	.07
Paralysis	19 (4.2%)	187 (1.9%)	.001
Psychosis	5 (1.0%)	152 (1.5%)	.57
Neurological disorders affecting movement	7 (1.5%)	173 (1.7%)	.87
Seizures and epilepsy	16 (3.5%)	141 (1.4%)	<.001
Solid organ transplant	9 (2.0%)	143 (1.4%)	.47
Dementia	6 (1.3%)	109 (1.1%)	.84
Lymphoma	2 (0.4%)	122 (1.2%)	.19
Drug abuse	1 (0.2%)	98 (1.0%)	.15
Acquired immune deficiency syndrome	7 (1.5%)	74 (0.7%)	.11
Solid tumor without metastasis, in situ	4 (0.8%)	54 (0.5%)	.54
Alcohol abuse	3 (0.6%)	22 (0.2%)	.17

Abbreviations: ICU, intensive care unit; IQR, interquartile range (25th–75th percentile); SD, standard deviation.

^a^Race in Epic Cosmos is self-reported according to the CDC categories [23].

^b^The SVI values reflect the CDC and Agency for Toxic Substances and Disease Registry (ATSDR) overall SVI percentile, which ranges from 0 to 1. Higher values indicate greater vulnerability [24]. Nine patients in the *C. auris* group and 278 in the non-*auris* group did not have information for this variable. The median and IQR were calculated for 448 and 9642 patients, respectively.

^c^Percentages in these categories are based on the total number of cases (N = 514 for the *C. auris* candidemia group and N = 10 386 for the group without a history of *C. auris*). Other percentages are calculated based on the total number of patients as per the CDC criteria (N = 457 in the *C. auris* group, N = 9920 in the non-*auris* group) [2].

^d^Percentages for the most recent discharge disposition are based on the total of cases with previous hospitalizations within 90 days of the index date (N = 282 for the *C. auris* candidemia group and N = 4120 for the group without a history of *C. auris*).

^e^Comorbidity data were unavailable for 2 patients in the *C. auris* group and 168 patients without a history of *C. auris* candidemia. Percentages are calculated based on the total number of patients (N = 457 in the *C. auris* group, N = 9920 in the non-*auris* group). Comorbidities were defined using the ECI from the International Classification of Diseases-10-Clinical Modification (ICD-10-CM) Refined Software [17]. The ECI score was calculated for each patient at the time of their first case of candidemia, and for the *C. auris* group, at the time of the first case of *C. auris* candidemia.

Age and gender did not differ significantly between patients with *C. auris* and non-*C. auris* candidemia (Table 2). In contrast, patients with *C. auris* were more likely to be Black (46.2% [211/457] vs 22.5% [2235/9920], *P* < .001). The social vulnerability index (SVI) (median 0.78 vs. 0.71, *P* < .001) and burden of comorbidities (mean ECI 15.9 ± 15.6 vs 13.2 ± 14.2, *P* < .001) were higher in patients with *C. auris*. Specific comorbidities overrepresented among patients with *C. auris* included severe renal failure (30.6% [140/457] vs 11.4% [1130/9920], *P* < .001), heart failure (23.8% [109/457] vs 14.1% [1405/9920], *P* < .001), obesity (17.5% [80/457] vs 11.1% [1102/9920], *P* < .001), and complicated diabetes (13.1% [60/457] vs 9.7% [967/9920], *P* = .02).

A higher proportion of recent hospitalization (54.9% [282/514] vs 39.7% [4120/10 386], *P* < .001) and discharge to nursing homes (41.8% [118/282] vs 19.6% [806/4120], *P* < .0001) and long-term care hospitals (12.4% [35/282] vs 2.2% [90/4120], *P* < .001) was observed among the *C. auris* cases. Although *C*. *auris* cases were less likely to be ICU-acquired (18.3% [94/514] vs 23.4% [2435/10 386], *P* = .008), they were more likely to have an ICU stay during hospitalization (59.3% [305/514] vs 51.7% [5375/10 386], *P* < .001). Thirty-day mortality was also similar (29.6% [152/514] vs 32.5% [3379/10 386], *P* = .17), as was in-hospital mortality (*P* = 1.0).

## DISCUSSION

In a nationwide electronic health record analysis of candidemia, we found that important epidemiologic trends in candidemia, including increases in incidence and 30-day mortality, rose sharply during the COVID-19 pandemic and persisted afterward. This increase in mortality was paralleled by a shift toward ICU-acquired cases and a trend toward older age. We additionally found that patients with *C. auris* were more likely to be Black and have high social vulnerability indices, renal failure, and recent healthcare exposure. Overall, these data suggest that candidemia is increasingly affecting medically vulnerable patients at high risk of mortality, such as the critically ill and elderly, and that important healthcare disparities exist with respect to *C. auris.*

In the prepandemic period, the 30-day mortality in our cohort was between 25% and 30%, aligning with previous CDC surveillance reports [2]. Consistent with CDC data, we found an increase in 30-day mortality during the COVID-19 pandemic, peaking at 37.6% in 2021, followed by a decline that remained above prepandemic levels [2]. Our analysis extends beyond the CDC report by including 2022–2024 and indicates a persistent increase in candidemia mortality. Overall, mortality from candidemia has continued to be high despite advances in prevention, diagnostics, and therapy. Host factors, including older age, comorbidities, and high-acuity illness, are associated with increased candidemia mortality and were prevalent in our cohort; they also increased over the past decade, which may have contributed to the persistently higher postpandemic mortality compared with prepandemic levels [25, 26]. However, our adjusted mortality model indicates that factors beyond those included (age, ECI score, COVID-19–related hospitalization, and ICU-acquired candidemia)—potentially including unmeasured patient characteristics and suboptimal management—may be driving the increased mortality and warrant further study.

In our data, *C. albicans* remained the most common species identified. However, its proportion decreased from 41.7% in 2015 to 36.4% in 2024. These findings contrast with CDC surveillance, which described a stable species distribution during 2015–2021 [2]. This may be due to our inclusion of the years 2022–2024. Indeed, we observed a rise in *C. auris* starting in 2019 and reaching 4.7% of candidemia cases by 2024. In the United States, information on patients with *C. auris* candidemia is limited [27–29], and few reports compare characteristics of *C. auris* with those of non-*C*. *auris* candidemia [27, 30–32]. Our unique nationwide study assesses specific risk factors for *C. auris* candidemia, finding several defining characteristics of *C. auris* candidemia that deserve specific mention. In our cohort, patients with *C. auris* were more likely to be Black and had higher SVI. The only previous US comparative cohort study comparing *C. auris* and non-*C*. *auris* cases was limited to three hospitals in New York City and identified age as a risk factor for *C. auris*, while sex and race did not differ, and SVI was not reported [27]. Non-US cohort studies did not report race or a social vulnerability marker [30–32].

Along with higher SVI, *C. auris* candidemia was associated with a higher comorbidity burden. We observed a clear relationship between *C. auris* and renal failure. An ICU cohort from India also reported an association of *C. auris* and chronic kidney disease (CKD) [31], and among patients with *C. auris* colonization, CKD has been reported as an independent factor for progression to candidemia [33]. In contrast, an association between CKD and *C. auris* was not observed in other cohorts [27, 30, 32]. CKD might be an independent risk factor for *C. auris* or a marker of indwelling device use or of broader healthcare exposure among patients with CKD. Regardless, this finding deserves further attention in follow-up studies, and dialysis centers and healthcare facilities caring for patients with CKD should consider heightened infection control practices for *C. auris*.

We additionally found that most patients with *C. auris* (54.9%) had recent hospitalizations, and many had recent discharges to nursing homes and long-term care hospitals. This reinforces prior smaller studies on the risk of *C. auris* outbreaks at these facilities and reinforces the need for heightened infection control [27]. While we could not assess whether there were institutional outbreaks at the acute care hospitals from which we drew our data, cases per health system (Supplementary Figure 30) indicate that *C. auris* candidemia cases were not limited to a small number of health systems. Importantly, while patients with *C. auris* had more comorbidities and a higher proportion of ICU stays during hospitalization, these factors were not accompanied by worse outcomes, and mortality was similar to that of other species. These findings are similar to prior studies [27, 30, 32] and suggest that *C. auris* may be less virulent than other species; further study here is warranted [34, 35].

From our antifungal susceptibility analysis, we found a rise in fluconazole resistance in *C. parapsilosis* and *C. tropicalis*, both common causes of candidemia [2]. For *C. parapsilosis* specifically, resistance increased from 2.5% in 2015 to 10.8% in 2024. This finding aligns with CDC surveillance, which reported 9.8% fluconazole resistance by 2021 [2]. Our results also align with another US study from 2015 to 2024, which showed increasing resistance, although with higher absolute rates (8.2% in 2015 to 20.3% in 2024) [36]. National-level data on fluconazole resistance in *C. tropicalis* are more limited. CDC surveillance based on 448 isolates showed year-to-year variability without a clear pattern [2]. In contrast, our study of 1872 isolates demonstrated an increase from 6.2% in 2015 to 18.1% in 2024, an important and concerning finding. These resistance trends for *C. parapsilosis* and *C. tropicalis* support the need for ongoing surveillance of azole resistance. Fortunately, echinocandin resistance was largely stable and rare.

This study has several limitations. The number of health systems contributing data increased over time, which could confound the results if populations significantly differed between health systems. However, the sensitivity analysis restricted to systems that consistently contributed data yielded results similar to those of our main analyses. Importantly, covariate balance (Supplementary Tables 5 and 6), temporal trends (Supplementary Table 7), and adjusted odds ratios for 30-day mortality (Supplementary Figures 24 and 25) were similar between sensitivity and main cohorts.

As an additional limitation, because postdischarge mortality must be transmitted to Epic Cosmos, 30-day mortality may be underestimated. We additionally analyzed in-hospital mortality to account for this potential limitation, and findings were similar to 30-day mortality. Next, comorbidities were identified using International Classification of Diseases (ICD)-10 codes listed by the Agency for Healthcare Research and Quality [17], and conditions may be missed if they were not in the standardized list. Regarding susceptibility, Epic Cosmos only reports categorical susceptibility interpretations derived from minimum inhibitory concentration (MIC) testing according to the CLSI-defined breakpoints [19], and actual MICs are not provided. As a result, susceptibility results may be affected by misclassification. To limit this, we analyzed only laboratory-verified isolates with valid interpretations (Supplementary Table 14). Further, reported susceptibility results reflect interpretations at the local laboratory and not reference laboratories; local practice may misclassify resistance [37, 38]. Moreover, the *C. auris* susceptibility data were available for only a subset of isolates, which may reflect variation in local laboratory practices and adoption of CDC tentative breakpoints introduced in 2019 [39]. Additionally, the clade classification of *C. auris* cases could not be assessed; antifungal resistance varies by clade [40]. Finally, we did not report some risk factors for candidemia, such as previous colonization, central venous catheters, and invasive procedures. In addition, previous outpatient procedures, surgeries, and hemodialysis could not be assessed; therefore, prior healthcare exposure may be underestimated. Reasons for admission and clinical course details, including the source of candidemia, source control, and antifungal therapy, could not be assessed. This is primarily due to limitations in Epic Cosmos; although important, these variables were outside the scope of this work.

In conclusion, our nationwide study of candidemia extending past the height of the COVID-19 pandemic demonstrates concerning trends, including increased mortality and high burden of *C. auris* among Black patients and patients with renal failure. Increased candidemia-related mortality was not fully explained by differences in age, comorbidities, concurrent COVID-19 infection, and ICU requirement. This study also highlights the need for antifungal resistance surveillance and testing in species with an increased rate of resistance to fluconazole, such as *C. parapsilosis* and *C. tropicalis.*

## Supplementary Material

ciag312_Supplementary_Data

## Supplementary Data

Supplementary materials are available at *Clinical Infectious Diseases* online. Consisting of data provided by the authors to benefit the reader, the posted materials are not copyedited and are the sole responsibility of the authors, so questions or comments should be addressed to the corresponding author.
