# Supplementary material for: Changes in Epidemiology of Candidemia in the United States With a Focus on Candida auris
Source: Clin Infect Dis. 2026 May 11;83(3):e505–14. doi: 10.1093/cid/ciag312 (PMC13644864; doi:10.1093/cid/ciag312)
Supplement: Supplementary file 1 [file ciag312_supplementary_data.docx]

**SUPPLEMENTARY MATERIALS**

Changes in Epidemiology of Candidemia in the United States with a Focus on *Candida auris*

**Contents**

[Authors	3](#_Toc221285912)

[METHODS	4](#_Toc221285913)

[Data modeling, variable definitions, bias, and mitigation strategies	4](#_Toc221285914)

[Sensitivity analysis	6](#_Toc221285915)

[Regression models	7](#_Toc221285916)

[Subset analysis	7](#_Toc221285917)

[Missing values	8](#_Toc221285918)

[SUPPLEMENTARY FIGURES	9](#_Toc221285919)

[Supplementary Figure 1. Distribution of candidemia patients by state identified from the Epic Cosmos database, 2015-2024	9](#_Toc221285920)

[Supplementary Figure 2. Mean age of patients with candidemia, 2015-2024	10](#_Toc221285921)

[Supplementary Figure 3. Race distribution of patients with candidemia, 2015-2024	11](#_Toc221285922)

[Supplementary Figure 4. Ethnicity distribution of patients with candidemia, 2015-2024	12](#_Toc221285923)

[Supplementary Figure 5. Gender distribution of patients with candidemia, 2015-2024	13](#_Toc221285924)

[Supplementary Figure 6. Candidemia incidence by year from 2015-2024, expressed as new cases per 100,000 hospital admissions	14](#_Toc221285925)

[Supplementary Figure 7. Onset location (community or health care) of candidemia cases, 2015-2024	15](#_Toc221285926)

[Supplementary Figure 8. Proportion of candidemia cases that were acquired in the intensive care unit, 2015-2024	16](#_Toc221285927)

[Supplementary Figure 9. Proportion of candidemia cases with an intensive care unit stay of at least one day during hospitalization	17](#_Toc221285928)

[Supplementary Figure 10. Proportion of candidemia cases with an intensive care unit stay of at least one day during the 13 days after the index date	18](#_Toc221285929)

[Supplementary Figure 11. In-hospital mortality after candidemia diagnosis, 2015-2024	19](#_Toc221285930)

[Supplementary Figure 12. Candidemia incidence among cases included in the sensitivity analysis (N=4,061) , expressed as new cases per 100,000 hospital admissions	20](#_Toc221285931)

[Supplementary Figure 13. Mean age of patients (N=3,821) with candidemia included in a sensitivity analysis of health systems that contributed data in all study years	21](#_Toc221285932)

[Supplementary Figure 14. Race of patients (N=3,821) with candidemia included in a sensitivity analysis of health systems that contributed data in all study years	22](#_Toc221285933)

[Supplementary Figure 15. Ethnicity of patients (N=3,821) with candidemia included in a sensitivity analysis of health systems that contributed data in all study years	23](#_Toc221285934)

[Supplementary Figure 16. Gender of patients (N=3,821) with candidemia included in a sensitivity analysis of health systems that contributed data in all study years	24](#_Toc221285935)

[Supplementary Figure 17. Community versus health care onset candidemia cases among cases (N=4,061) included in a sensitivity analysis of health systems that contributed data in all study years	25](#_Toc221285936)

[Supplementary Figure 18. Proportion of candidemia cases that were acquired in the intensive care unit among cases (N=4,061) included in a sensitivity analysis of health systems that contributed data in all study years	26](#_Toc221285937)

[Supplementary Figure 19. Proportion of candidemia cases with an intensive care unit stay of at least one day during hospitalization among cases (N=4,061) included in a sensitivity analysis of health systems that contributed data in all study years	27](#_Toc221285938)

[Supplementary Figure 20. Proportion of candidemia cases with an intensive care unit stay of at least one day during the 13 days after the index date among cases (N=4,061) included in a sensitivity analysis of health systems that contributed data in all study years	28](#_Toc221285939)

[Supplementary Figure 21. 30-day mortality by candidemia cases among cases (N=4,061) included in a sensitivity analysis of health systems that contributed data in all study years	29](#_Toc221285940)

[Supplementary Figure 22. In-hospital mortality by candidemia case admission among cases (N=4,061) included in a sensitivity analysis of health systems that contributed data in all study years	30](#_Toc221285941)

[Supplementary Figure 23. Proportion of Candida spp. per candidemia cases among cases (N=4,061) included in a sensitivity analysis of health systems that contributed data in all study years	31](#_Toc221285942)

[Supplementary Figure 24. Adjusted odds ratio for 30-day mortality in the sensitivity cohort	32](#_Toc221285943)

[Supplementary Figure 25. Adjusted odds ratios for 30-day mortality over time: Primary vs sensitivity analysis	33](#_Toc221285944)

[Supplementary Figure 26. 30-day mortality according to Candida species, 2015 – 2024	34](#_Toc221285945)

[Supplementary Figure 27. Rate of fluconazole resistance among Candida species by CDC-defined geographic regions of the United States	35](#_Toc221285946)

[Supplementary Figure 28. Rate of resistance to any echinocandin (micafungin, anidulafungin, or caspofungin) among Candida species by CDC-defined geographic regions of the United States	36](#_Toc221285947)

[Supplementary Figure 29. Distribution of Candida auris patients (N = 476) by state identified from the Epic Cosmos database, 2015-2024	37](#_Toc221285948)

[Supplementary Figure 30. Health systems contributing to the C. auris dataset: Annual incident case counts of C. auris.	38](#_Toc221285949)

[SUPPLEMENTARY TABLES	39](#_Toc221285950)

[Supplementary Table 1. Distribution of candidemia cases per patient and species	39](#_Toc221285951)

[Supplementary Table 2. Distribution of species in recurrence cases (all cases following the initial case)	40](#_Toc221285952)

[Supplementary Table 3. Total candidemia cases per year, annual incidence, and change in incidence	41](#_Toc221285953)

[Supplementary Table 4. Demographics, risk factors, and outcomes among N=3,821 patients with candidemia identified from Epic Cosmos, 2015-2024, and included in a sensitivity analysis	42](#_Toc221285954)

[Supplementary Table 5. Standardized Mean Differences (SMDs) for Covariate Balance: Full vs Sensitivity Cohort	44](#_Toc221285955)

[Supplementary Table 6. Standardized Mean Differences (SMDs) for Covariate Balance Year by Year in outcomes	46](#_Toc221285957)

[Supplementary Table 7. Temporal Concordance Analysis Between Full Cohort and Subset Populations from 2015-2024	48](#_Toc221285959)

[Supplementary Table 8. Antifungal resistance of Candida albicans isolates, 2015-2024	50](#_Toc221285961)

[Supplementary Table 9. Antifungal resistance of Nakaseomyces glabratus isolates, 2015-2024	51](#_Toc221285962)

[Supplementary Table 10. Antifungal resistance of Pichia kudriavzevii isolates, 2015-2024	52](#_Toc221285963)

[Supplementary Table 11. Antifungal resistance of Candida parapsilosis isolates, 2015-2024	53](#_Toc221285964)

[Supplementary Table 12. Antifungal resistance of Candida tropicalis isolates, 2015-2024	54](#_Toc221285965)

[Supplementary Table 13. Antifungal resistance of Candida auris isolates, 2022-2024	55](#_Toc221285966)

[Supplementary Table 14. The total number of Candida species isolates with missing or invalid antifungal susceptibility results that were excluded from the analysis	56](#_Toc221285967)

[REFERENCES	57](#_Toc221285968)

# **Authors**

Maria Alejandra Perez, MD, Khush A. Patel, MD, MS, Laila Rasmy, PhD, Hisato Yoshida DDS, PhD, Cesar A. Arias, MD, PhD, Masayuki Nigo, MD, MSc, Max W. Adelman, MD, MSc

# **METHODS**

## **Data modeling, variable definitions, bias, and mitigation strategies**

This is a retrospective cohort study using the Epic Cosmos database.^7^ Epic Cosmos is a dataset created in collaboration with health systems using the Epic electronic health record system, representing more than 300 million patient records from over 1,809 hospitals and 41,400 clinics as of October 2025. This section explains in detail the process of constructing the main database, including the steps of data extraction and data cleaning to address possible bias. For clarity, the definitions of the variables in Epic Cosmos are provided. Since Epic Cosmos organizes information into multiple tables, the original names of these tables will be used in this text to facilitate reproducibility.

Using R software version 4.4.1,^8^ we identified the tables “Lab susceptibility result fact” and “Lab organism dim”, which store data about microbiology isolate and susceptibility results. Together, they formed our initial dataset, which lists the specimen source, collection date, organism genus and species, antifungals tested, interpretation, and a patient identification (ID) number. Using an initial query on these tables, we identified positive blood cultures for the target genera from 2015–2024, as detailed in the main article. We removed the isolates that the laboratory did not verify and those with suppressed susceptibility results. Given that Epic Cosmos updates continuously, we fixed the dataset on August 23, 2025.

Using patient IDs from the original dataset, we pulled demographics from the table “Patient dim”; hospital admissions from “Hospital admission fact”; health care and intensive care unit (ICU) encounters from “Encounter fact” and “ICU stay registry data mart”; prior diagnoses from “Diagnosis event fact,” “Diagnosis terminology dim,” and “Diagnosis set dim”; and COVID-19 history from “Covid patient data mart X.”

The “Patient dim” table holds one up-to-date record of the demographic characteristics per patient. As definitions, race and ethnicity are described in Epic Cosmos as self-reported by the patient, and Epic standardizes both to CDC codes^2^. Sex is defined as the legal sex recorded by a government body. We treated race, ethnicity, and legal sex as stable over time during each patient’s case. As for age, Epic Cosmos shifts dates to protect birthdate privacy while supporting quality reporting. Using the provided birth date, we computed age at each case’s index date.

Following the demographic variables, Epic Cosmos reports the CDC Social Vulnerability Index (SVI) 2020.^3^ The CDC publishes SVI at the census tract level, a predefined and stable geographical area unit. Epic Cosmos assigns a Federal Information Processing Standards code based on the latitude and longitude of the patient’s address and links the corresponding tract-level SVI value. In Epic Cosmos, contributing hospitals are de-identified and grouped under an ID that may represent one or more hospitals; these groups are referred to as health systems or “SourceKeys” in Epic Cosmos.^9^ Because geographic location cannot be determined from the health system ID, state-level contributions shown in **Supplementary Figure 1** and **Supplementary** **Figure 29** were mapped using the patient’s reported state in the “Patient dim” table, in which both SVI and state are derived from the most recent address recorded. As a result, they may not reflect the patient’s address at the time of the candidemia episode, introducing potential information bias. Because these demographic characteristics exist only in “Patient dim”, cross-validation with other Epic Cosmos tables was not possible. The variables described before were joined to the initial dataset using the patient ID.

Epic Cosmos captures mortality information in two tables, one of which is the “Patient dim” table. In this table, the date of death is derived from the patient's chart or the most recent load from external sources, such as other provider organizations and national exchange networks. To reduce misclassification bias, we also used the discharge disposition field “expired” from the “Encounter fact” table to confirm that the patient had deceased. Using these two sources increases the specificity of in-hospital mortality classification but may reduce sensitivity for deaths occurring after discharge or outside the hospital. Importantly, results using in-hospital mortality were largely consistent with those observed for 30-day mortality, suggesting that missing some deaths outside the hospital is unlikely to have changed our overall conclusions.

As part of database construction, we pulled admission and discharge dates from “Hospital admission fact” and “Encounter fact” tables. In Epic Cosmos, an encounter is any documented patient interaction with the health care system. We excluded encounters with incomplete dates, then combined consecutive encounters when the admission date followed the prior discharge date on the same day or within one day. This approach limited overcounting from internal transfers and produced our hospital-stay windows. By doing so, we aimed to reduce the information bias related to incorrect time aggregation. Finally, we assigned to each case the hospitalization whose stay window included the index date. Similarly, the same steps were followed in the “ICU stay registry data mart” table to define ICU stay intervals at the case level. Then, the established hospital- and ICU-stay windows were joined to the initial dataset by patient ID and used to classify case onset, ICU stay during hospitalization, ICU stay during the 13 days after the index date, and in-hospital mortality.

As a limitation, we could not evaluate certain recent health care–related exposures (e.g., surgery or hemodialysis) needed to fully apply the CDC definition of health care–associated community onset.^4^ However, hospitalization in the previous 90 days before the index date was assessed. This was addressed using the same methodology previously described to produce the hospital-stay windows. It should be noted that Epic Cosmos does not capture patients’ place of origin at the time of hospital admission (e.g., home, nursing home, or a long-term care hospital). However, it does include a discharge disposition field in the “Encounter Fact” and “Hospital Admission Fact” tables. Using the most recent hospitalization prior to the candidemia index-date hospitalization window, we evaluated discharge disposition to identify patients whose most recent discharge was to a nursing home or a long-term care hospital.

We built the comorbidity dataset using information from three tables: “Diagnosis event fact,” “Diagnosis terminology dim,” and “Diagnosis set dim”. We analyzed all types of diagnoses, including those recorded in outpatient encounters and hospital stays. Epic Cosmos sets the start date to the date the diagnosis event begins; we included only diagnoses with a start date available, and only if that date preceded each case index date, to document past medical history. In addition, to improve the data quality, we kept only diagnoses with an identified health system and codes labeled as “trusted” in Epic Cosmos (“trusted” indicates the record met Epic Cosmos data-quality criteria and is suitable for reporting).

In Epic Cosmos, the diagnoses are coded in standard terminologies that include the International Classification of Diseases (ICD) and the Systematized Nomenclature of Medicine – Clinical terms (SNOMED-CT). We assigned each diagnosis code to a comorbidity group using the Agency for Healthcare Research and Quality (AHRQ) ICD-10 mapping.^5^ We excluded SNOMED-CT codes because no standard list exists to give a consistent and reproducible identification of comorbidities groups using that terminology. We manually added solid organ transplant (SOT) codes, as they were not included in the reference list but represent a population at risk for candidemia. We identified SOT codes from the ICD-10 website^10^, and those included are: 'Z94.0', 'V42.0', 'Z48.22', 'Z94.4', 'V42.7', 'Z48.23', 'Z94.2', 'V42.6', 'Z48.24', 'Z94.1', 'V42.1', 'V42.2', 'Z48.21', 'V42.83', 'Z94.83', 'V42.84', 'Z94.82', 'V42.9', 'Z48.290'. This approach may produce misclassification bias because ICD-10 codes for some comorbidities that are not in the AHRQ list or in our SOT list are missed. Additionally, the recorded diagnoses depend on the information provided in the medical records, and there may be a source bias if any errors occurred in the documentation. Following the AHRQ model, we calculated the Elixhauser Comorbidity Index (ECI), applying the scores for mortality risk.^5^

We used the “Covid patient data mart X” table to identify recent COVID-19 diagnoses at admission or during the hospital stay. COVID-19 positivity was defined as the presence of a date in the “Covid positive date Key” field. This field stores the earliest date a patient is considered COVID-19 positive based on any of the following: a documented diagnosis (encounter diagnosis, billing diagnosis, or hospital problem), a positive COVID-19 test, or if the first positive test occurred after the recorded death, the death date. That field was added to the initial dataset using the patient ID. We defined a COVID-19–related hospitalization as a COVID-19 diagnosis made within 7 days before admission or at any time during the hospitalization,^9^ at the case level.

As part of the statistical analysis, incidence was estimated as new cases of candidemia, with hospital admissions selected as the denominator. To estimate the population at risk within the same health systems contributing candidemia cases, we identified the corresponding Health System IDs in the “Hospital Admissions Fact” table. Then, to avoid overcounting admissions, we used the same hospital-stay window approach described previously to group related encounters into a single hospitalization and assign unique identifiers to count distinct hospital admissions per year. In addition, to further minimize overestimation of the denominator, annual admissions for each health system were counted beginning in the first year that the health system contributed candidemia cases to the “Lab Susceptibility Result Fact” table. This approach was used to align the denominator with the period during which laboratory-confirmed candidemia cases were observable in Epic Cosmos. The final incidence measure was expressed as new candidemia cases per 100,000 hospital admissions.

For antifungal susceptibility analysis, Epic Cosmos does not provide minimum inhibitory concentration (MIC) values of each antifungal; instead, the susceptibility interpretative categories of the MIC values reported by laboratory staff are stored. To handle the source bias, we included only isolates with a laboratory-verified mark and only isolates with valid interpretations. These valid interpretations are defined as those established by the Clinical and Laboratory Standards Institute (CLSI) or CDC and include susceptible, susceptible-dose dependent, intermediate, resistant, or non-susceptible (considered equivalent to resistant).^11,12^ We excluded antifungal report results with missing or empty values or invalid interpretations such as “unspecified,” “moderately susceptible,” or “very susceptible.” However, possible biases that cannot be controlled include information bias and the risk of misclassification. For all the antifungal susceptibility analyses, only species with CLSI or CDC-defined breakpoints to these antifungals were included.^11,12^

The resistance rates to fluconazole and echinocandins by region were estimated following the CDC geographic regions of the U.S.^1^ As noted above, because the health system identifier does not provide geographic information, resistance patterns were mapped using the patient’s reported state from demographic records. Isolates reported as resistant but without a specified state were excluded from this geographical analysis. For transparency, we report not only resistance rates but also the total number of isolates tested, and the number found resistant (**Supplementary figures 27-28**). However, this analysis may result in misclassification bias because the isolate could be assigned to the wrong state if the patient moved or received care in a different location than the most recent record in the demographics table.

## **Sensitivity analysis**

We conducted a sensitivity analysis by limiting the sample to health systems that met two predefined criteria to ensure that trends were evaluated over time within a consistent set of health systems and to improve data quality for the antifungal susceptibility analysis. The first criterion for data quality was consistent contribution of hospital and ICU records to Epic Cosmos from 2015 to 2024. For this, the number of hospital admission records in the last decade was counted for each health system (using the “Hospital admission fact” table); then, using a Pareto cut, the health systems contributing more than 80% of the admission records and that contributed consistently across 2015–2024 were selected. We used the same approach to identify health systems with sustained data reporting in the “ICU Stay Registry Data Mart” table. Then, only health systems present in both searches were mapped.

The second criterion was reliable susceptibility interpretations for *Candida* species isolated in blood cultures. For this, only the health systems in which the main clinically relevant species and antifungals of interest had fewer than 5% missing values or invalid interpretations were selected (using the “Lab susceptibility result fact” table). This step was used to reduce information bias due to nonstandard susceptibility reporting. The selected species for this filter were the most common in our analysis and included: *C. albicans, N. glabratus, C. parapsilosis, C. tropicalis, C. auris, P. kudriavzevii, C. lusitaniae*, and *C. dubliniensis.* The antifungals of interest included fluconazole, voriconazole, anidulafungin, caspofungin, and amphotericin B.

In addition to those primary criteria, we considered including data completeness in the specific tables and fields used as a criterion. In this approach, for each health system, we measured the field-level of missingness across its encounters from 2015 to 2024 in the complete Epic Cosmos database (treating “−1” in Epic Cosmos fields as missing). Specifically, in the “Hospital Admission Fact” table (contributing health systems, N=270), the Admission Date Key field showed 0% of health systems with >5% of missing values, whereas the Discharge Date Key field had 13% (35/270) of systems with >5% missingness. In the “ICU Stay Registry Data Mart” (N=267), both ICU Stay Start Instant and ICU Stay End Instant had 0% of health systems with more than 5% missingness. In the comorbidities dataset (N=309), the Start Date Key field had 0% with >5% missingness, while the Diagnosis Key exceeded 5% missingness in 4.5% (14/309) of systems. By contrast, standardized, valid antifungal interpretations in the “Lab Susceptibility Result Fact” table provided a more informative quality measure for identifying systems with reliable reporting and elected to use this as a key determinant of data quality.

After applying the two criteria, we identified 13 eligible health systems from Epic Cosmos that met the above data quality thresholds. Then, using the health system IDs as keys, we repeated the previously described process to assemble a new dataset with the same structure as the original. We processed it with the same cleaning steps and statistical methods.

To assess how the restricted sensitivity cohort differed from the full cohort, we compared baseline characteristics using standardized mean differences (SMDs). For categorical variables, we calculated proportions using the appropriate denominator (patient or case totals, as specified for each variable in the Table 1 footnotes) and estimated SMDs as the difference in proportions divided by the pooled standard deviation of the proportions. Balance was summarized using absolute SMD thresholds (<0.1 good, 0.1–0.25 acceptable, ≥0.25 poor), and variables were ranked by absolute SMD to identify the largest cohort differences.^13,14^ Standardized mean differences were calculated using the R package cobalt.

For each variable, we aligned the full and subset annual series to years and quantified concordance in temporal patterns using Spearman’s rank correlation (ρ) and its p-value. We fit linear regression models by ordinary least squares, including a year x cohort interaction, to test whether temporal slopes differed between cohorts (p < 0.05 interpreted as evidence of different slopes; p ≥ 0.05 as no evidence of different slopes, consistent with parallel trends). Trend direction was summarized using the sign of the year slopes from the same model. Spearman’s rank correlation (ρ) and its p-value were computed using cor.test (method = “spearman”), and differences in temporal slopes between cohorts were assessed using lm with a year×cohort interaction.^6^

## **Regression models**

Using the full cohort of all health systems and all patient episodes, we estimated 30-day mortality using a multivariable logistic regression model that included age, Elixhauser comorbidity index, COVID-19–related hospitalization, ICU-acquired bloodstream infection, and calendar year (as a categorical variable). We added a fixed-effects intercept for health systems to control for stable differences across systems. This approach focuses the year-to-year estimates on changes within the same health system and helps reduce bias from changes in the set of contributing health systems across the study period. Because patients could contribute more than one episode and observations within a health system are correlated, we used two-way cluster-robust standard errors at the patient and health-system levels.^9^ The model was fit using feglm from the fixest package in R.

In this model, 2017 is the reference year because its adjusted mortality was closest to the median adjusted mortality across pre-pandemic years (0.26).

A random-intercepts model was not selected for the primary analysis because it assumes that unobserved baseline differences between systems are not correlated with calendar time; this assumption may be compromised when health system participation changes over time (e.g., increasing representation of larger, higher-acuity tertiary centers in later years).

For the sensitivity analysis of 30-day mortality, we limited observations to each patient’s first candidemia episode in the restricted subset of 13 health systems that contributed data consistently during the study period. In this restricted cohort, we fit a mixed-effects logistic regression with a random intercept by health system.^9^ In this subset, 2018 was used as the reference year because its adjusted mortality was closest to the median adjusted mortality across pre-pandemic years (0.25). We used a random-intercepts specification in this analysis because it allows each system to have its own baseline mortality level and accounts for clustering within systems, which can yield more stable estimates in this restricted dataset. This model was fit using glmer from the lme4 package.

## **Subset analysis**

We compared patients with *C. auris* candidemia to those with non–*C. auris* candidemia. To construct the *C. auris* group, we identified patients with a record of a case attributed exclusively to *C. auris* from the assembled dataset, analyses were restricted to those *C. auris* cases. Then we identified the health systems that reported these *C. auris* cases (39 systems) and drew a comparison group from the same systems, restricted to the period when *C. auris* occurred (2019–2024), consisting of patients with non–*C. auris* candidemia and no documented history of *C. auris* infection.

Demographic characteristics and comorbidities were evaluated at the patient level. For the *C. auris* group, ECI and mapped comorbidity groups were based on diagnosis codes recorded before the index date of the first *C. auris* case. For the non*-C. auris* group, at the time of the first case index date. As with the original cohort, we analyzed outcomes, COVID-10 related hospitalization and infection onset at the case level.^4^

For the comparison between groups, categorical variables were tested using the Chi-square test (chisq.test function). For numerical variables, normality was assessed with the Lilliefors test (lillie.test function), a variation of the Kolmogorov–Smirnov test.^15^ If both groups were normally distributed, a t-test (t.test function) was applied; otherwise, the Wilcoxon rank-sum test (wilcox.test function) was used.

## **Missing values**

In the demographic analyses (original and sensitivity dataset), the missing values were reported in the descriptive tables; since they accounted for <5% of observations in some of the variables, no imputation method was applied. For the susceptibility analysis, isolates with missing or invalid interpretations were excluded (summarized in **Supplementary Table 14**). Because the missing values accounted for >20% of observations for most *Candida species*, no imputation was performed. We report resistance rates only for isolates with valid interpretations based on CLSI and CDC interpretations^11,12^; this reduces the information and analytical bias that might result in false resistance assignments in an imputation method with high missingness.

# **SUPPLEMENTARY FIGURES**

**Supplementary Figure 1. Distribution of candidemia patients by state identified from the Epic Cosmos database, 2015-2024**

A total of 507 patients did not have a specified state, or the state was masked in the database.

## **Supplementary Figure 2. Mean age of patients with candidemia, 2015-2024**

Mean age increased over time (p=0.0007 by Mann-Kendall test). Abbreviations: SD = standard deviation.

## **Supplementary Figure 3. Race distribution of patients with candidemia, 2015-2024**

The proportion of Black patients increased over time (p=0.004), and the proportion of white patients decreased (p=0.0007, by the Mann-Kendall test). 520 patients with unspecified race were not included in this figure but included in the denominator for calculating the percentage.

## **Supplementary Figure 4. Ethnicity distribution of patients with candidemia, 2015-2024**

The proportion of Hispanic/Latino patients increased over time (p=0.004 by Mann-Kendall test). Ethnicity was unspecified for 1203 patients (4.3% of the total patients). Abbreviations: n = number of patients.

## **Supplementary Figure 5. Gender distribution of patients with candidemia, 2015-2024**

There was no significant change in gender distribution over time (p= 0.10 by Mann-Kendall test). Gender was unknown for 17 patients. Abbreviations: n = number of patients.

## **Supplementary Figure 6. Candidemia incidence by year from 2015-2024, expressed as new cases per 100,000 hospital admissions**

Incidence increased over time (p=0.02 by Mann-Kendall test). Overall incidence across the study period was 50.5 per 100,000 hospital admissions.

**Supplementary Figure 7. Onset location (community or health care) of candidemia cases, 2015-2024**

There was no significant trend in onset location over the study period by Mann-Kendall test (p=0.07). Abbreviations: n = number of cases, BSI = Bloodstream infection.

## **Supplementary Figure 8. Proportion of candidemia cases that were acquired in the intensive care unit, 2015-2024**

The proportion increased over time (p=0.03 by Mann-Kendall test). Abbreviations: n = number of cases.

## **Supplementary Figure 9. Proportion of candidemia cases with an intensive care unit stay of at least one day during hospitalization**

The proportion increased over the study period (p=0.05 by Mann-Kendall test). Abbreviations: n = number of cases.

## **Supplementary Figure 10. Proportion of candidemia cases with an intensive care unit stay of at least one day during the 13 days after the index date**

The proportion increased over the study period (p=0.05 by Mann-Kendall test). Abbreviations: n = number of cases.

## **Supplementary Figure 11. In-hospital mortality after candidemia diagnosis, 2015-2024**

In-hospital mortality increased over the study period (p=0.02 by Mann-Kendall test). Abbreviations: n = number of cases.

## **Supplementary Figure 12. Candidemia incidence among cases included in the sensitivity analysis (N=4,061), expressed as new cases per 100,000 hospital admissions**

Incidence increased over the study period (p=0.007 by Mann-Kendall test). Overall incidence across the study period was 57.4 per 100,000 hospital admissions.

## **Supplementary Figure 13. Mean age of patients (N=3,821) with candidemia included in a sensitivity analysis of health systems that contributed data in all study years**

Mean age increased over the study period (p=0.007 by Mann-Kendall test). Abbreviations: n = number of cases. Abbreviations: SD = standard deviation.

## **Supplementary Figure 14. Race of patients (N=3,821) with candidemia included in a sensitivity analysis of health systems that contributed data in all study years**

There was no significant trend in proportion of white (p=0.12) or Black (p=0.28) patients over time.

## **Supplementary Figure 15. Ethnicity of patients (N=3,821) with candidemia included in a sensitivity analysis of health systems that contributed data in all study years**

There was no significant trend in the proportion of Hispanic/Latino (p=0.28) patients over time. Abbreviations: n = number of patients.

## **Supplementary Figure 16. Gender of patients (N=3,821) with candidemia included in a sensitivity analysis of health systems that contributed data in all study years**

There was no significant change in proportion of male patients (p=0.72) over time. Abbreviations: n = number of patients.

## **Supplementary Figure 17. Community versus health care onset candidemia cases among cases (N=4,061) included in a sensitivity analysis of health systems that contributed data in all study years**

There was no significant change in the proportion of health care onset cases over the study period (p=0.15). Abbreviations: n = number of cases.

## **Supplementary Figure 18. Proportion of candidemia cases that were acquired in the intensive care unit among cases (N=4,061) included in a sensitivity analysis of health systems that contributed data in all study years**

There was no significant trend in ICU-acquired cases (p=0.21). Abbreviations: n = number of cases.

## **Supplementary Figure 19. Proportion of candidemia cases with an intensive care unit stay of at least one day during hospitalization among cases (N=4,061) included in a sensitivity analysis of health systems that contributed data in all study years**

The proportion of patients who required ICU stay increased over the study period (p=0.04). Abbreviations: n = number of cases.

## **Supplementary Figure 20. Proportion of candidemia cases with an intensive care unit stay of at least one day during the 13 days after the index date among cases (N=4,061) included in a sensitivity analysis of health systems that contributed data in all study years**

There was no significant trend over the study period (p=0.37). Abbreviations: n = number of cases.

## **Supplementary Figure 21. 30-day mortality by candidemia cases among cases (N=4,061) included in a sensitivity analysis of health systems that contributed data in all study years**

There was no significant increase in 30-day mortality in the sensitivity analysis (p=0.07). Abbreviations: n = number of cases.

## **Supplementary Figure 22. In-hospital mortality by candidemia case admission among cases (N=4,061) included in a sensitivity analysis of health systems that contributed data in all study years**

There was no significant trend over the study period (p=0.10). Abbreviations: n = number of cases.

## **Supplementary Figure 23. Proportion of *Candida* spp. per candidemia cases among cases (N=4,061) included in a sensitivity analysis of health systems that contributed data in all study years**

## **Supplementary Figure 24. Adjusted odds ratio for 30-day mortality in the sensitivity cohort**

Sensitivity analysis restricted to each patient’s first candidemia episode within the 13 health systems contributing data in all study years. Points show adjusted odds ratios (aORs) for 30-day mortality by year from a mixed-effects logistic regression with a health-system–specific random intercept. Shaded band indicates 95% confidence intervals. Year 2018 was the reference.

## **Supplementary Figure 25. Adjusted odds ratios for 30-day mortality over time: Primary vs sensitivity analysis**

Year-specific adjusted odds ratios (aORs) for 30-day mortality from the primary and sensitivity analyses. The primary analysis in the full cohort used multivariable logistic regression including age, Elixhauser comorbidity index, COVID-19–related hospitalization, ICU-acquired bloodstream infection, and calendar year (categorical), with health-system fixed effects and two-way cluster-robust standard errors at the patient and health-system levels. The sensitivity analysis was restricted to each patient’s first candidemia episode within the 13 health systems contributing data in all study years and was estimated using mixed-effects logistic regression with a health-system–specific random intercept. Temporal trends were broadly similar in direction in the primary and sensitivity analyses.

## **Supplementary Figure 26. 30-day mortality according to *Candida* species, 2015 – 2024**

30-day mortality due to *C. albicans* and *C. parapsilosis* increased over the study period (Mann–Kendall p = 0.007 and p = 0.01, respectively), while other species showed no significant trends.

**Supplementary Figure 27. Rate of fluconazole resistance among *Candida* species by CDC-defined geographic regions of the United States^1^**

Percentages represent the proportion of fluconazole-resistant isolates relative to the total number of isolates tested in each region. From the selected species, a total of 320 isolates that did not have a state were excluded from the analysis.

**Supplementary Figure 28. Rate of resistance to any echinocandin (micafungin, anidulafungin, or caspofungin) among *Candida* species by CDC-defined geographic regions of the United States^1^**

Percentages represent the proportion of echinocandin-resistant isolates relative to the total number of isolates tested in each region. From the selected species, a total of 287 isolates that did not have a state were excluded from the analysis.

**Supplementary Figure 29. Distribution of *Candida auris* patients (N = 476) by state identified from the Epic Cosmos database, 2015-2024**

## **Supplementary Figure 30. Health systems contributing to the *C. auris* dataset: Annual incident case counts of *C. auris***

Health system IDs were assigned numeric codes to de-identify the original system identifiers. *Indicates no candidemia reports in that year (including both *C. auris* and non–*C. auris* candidemia cases).

# **SUPPLEMENTARY TABLES**

## **Supplementary Table 1. Distribution of candidemia cases per patient and species**

| **Variable** | **N** | **%** | **Mean (SD)** | **Median (IQR)** | **Range** |
| --- | --- | --- | --- | --- | --- |
| Patients | 27,926 |  |  |  |  |
| Cases, total | 29,454 |  |  |  |  |
| Cases per patient |  |  | 1.05 (0.32) | 1 (1-1) | 1-16 |
| 1 | 26,816 | 96.0% |  |  |  |
| 2 | 895 | 3.2% |  |  |  |
| 3 | 139 | 0.5% |  |  |  |
| ≥4 | 76 | 0.3% |  |  |  |
| Cases per species^a^ |  |  |  |  |  |
| *Candida albicans* | 11,782 | 40.0% |  |  |  |
| *Nakaseomyces glabratus* | 9,462 | 32.1% |  |  |  |
| *Candida parapsilosis* | 3,981 | 13.5% |  |  |  |
| *Candida tropicalis* | 2,203 | 7.5% |  |  |  |
| *Candida dubliniensis* | 665 | 2.3% |  |  |  |
| *Pichia kudriavzevii* | 577 | 2.0% |  |  |  |
| *Candida auris* | 533 | 1.8% |  |  |  |
| *Clavispora lusitaniae* | 500 | 1.7% |  |  |  |
| *Candida guilliermondii* | 148 | 0.5% |  |  |  |
| *Candida kefyr* | 72 | 0.2% |  |  |  |
| *Candida metapsilosis* | 50 | 0.2% |  |  |  |
| *Candida orthopsilosis* | 50 | 0.2% |  |  |  |
| Other species^b^ | 170 | 0.4% |  |  |  |

^a^ Some cases involved >1 species, so the proportion adds to >100%. The denominator used to calculate the proportion was the total number of cases (N=29,454).

^b^Other species included: unspecified, 43 (0.1%); *C. rugosa*, 25 (0.1%); *C. lipolytica*, 23 (0.1%); *C. pelliculosa*, 20 (0.1%); *C. utilis*, 12 (0.0%); *C. famata*, 8 (0.0%); *C. lambica*, 6 (0.0%); *C. norvegensis*, 6 (0.0%); *Kluyveromyces marxianus*, 6 (0.0%); *C. haemulonii*, 5 (0.0%); *C. catenulata*, 4 (0.0%); *C. intermedia*, 2 (0.0%); *C. sphaerica*, 2 (0.0%); *C. bracarensis*, 1 (0.0%); *C. ciferrii*, 1 (0.0%); *C. colliculosa*, 1 (0.0%); *C. duobushaemulonii*, 1 (0.0%); *C. inconspicua*, 1 (0.0%); *C. pararugosa*, 1 (0.0%); *C. pulcherrima*, 1 (0.0%); and *Kluyveromyces* (unspecified), 1 (0.0%).

## **Supplementary Table 2. Distribution of species in recurrence cases (all cases following the initial case)**

| **Species** | **Number of cases** | **%**  **(N=1,471)** |
| --- | --- | --- |
| *Candida albicans* | 431 | 29.30% |
| *Nakaseomyces glabratus* | 431 | 29.30% |
| *Candida parapsilosis* | 392 | 26.65% |
| *Candida tropicalis* | 111 | 7.51% |
| *Candida auris* | 75 | 5.10% |
| *Pichia kudriavzevii* | 32 | 2.18% |
| *Candida Dubliniensis* | 30 | 2.04% |
| *Clavispora lusitaniae* | 18 | 1.22% |
| *Candida Guilliermondii* | 12 | 0.82% |
| *Candida metapsilosis* | 9 | 0.61% |
| *Other species^b^* | 9 | 0.61% |
| *Candida orthopsilosis* | 5 | 0.34% |
| *Candida kefyr* | 3 | 0.20% |

Among 1,110 patients with ≥2 documented cases, recurrent cases occurring after each patient’s index case totaled 1,471 and are analyzed in this table.

^b^Other species included: unspecified, 3 (0.2%); *C. rugosa*, 1 (0.07%); *C. lipolytica*, 2 (0.14%); *C. pelliculosa*, 3 (0.20%).

## **Supplementary Table 3. Total candidemia cases per year, annual incidence, and change in incidence**

| **Year** | **Candidemia cases (N=29,454)** | **Hospital admissions (N=58,305,784)** | **Candidemia incidence (new cases per 100,000 hospital admissions)** | **Incidence change from the year prior (%)** |
| --- | --- | --- | --- | --- |
| 2015 | 1,024 | 2,418,895 | 42.3 | N/A |
| 2016 | 1,370 | 3,053,919 | 44.9 | +6.1 |
| 2017 | 1,856 | 3,852,927 | 48.2 | +7.3 |
| 2018 | 2,198 | 4,774,551 | 46.0 | -4.6 |
| 2019 | 2,585 | 5,350,861 | 48.3 | +5.0 |
| 2020 | 2,998 | 5,655,164 | 53.0 | +9.7 |
| 2021 | 3,734 | 6,943,962 | 53.8 | +1.5 |
| 2022 | 4,052 | 7,608,997 | 52.3 | -2.8 |
| 2023 | 4,574 | 8,759,703 | 52.2 | -0.2 |
| 2024 | 5,063 | 9,886,805 | 51.2 | -1.9 |
| Overall | 29,454 | 58,305,784 | 50.5 |  |

**Supplementary Table 4. Demographics, risk factors, and outcomes among N=3,821 patients with candidemia identified from Epic Cosmos, 2015-2024, and included in a sensitivity analysis**

| **Variable** | **Value** |
| --- | --- |
| Age, years, mean (SD) | 58.0 (19.2) |
| Age ≥ 18 years | 3,688 (96.5%) |
| Sex, N (%) |  |
| Female | 1,663 (43.5%) |
| Male | 2,158 (56.5%) |
| Unknown | 0 |
| Race, N (%)^a^ |  |
| American Indian/Alaska Native | 25 (0.6%) |
| Asian | 58 (1.5%) |
| Black/African American | 853 (22.3%) |
| Native Hawaiian/Pacific Islander | 5 (0.1%) |
| Other | 78 (2.0%) |
| White | 2,783 (72.8%) |
| Ethnicity |  |
| Not Hispanic/Latino | 3,447(90.2%) |
| Hispanic/Latino | 265 (6.9%) |
| Unspecified | 109 (2.8%) |
| Social Vulnerability Index, overall percentile, median (IQR)^b^ | 0.65 (0.42 – 0.90) |
| Onset^c^ |  |
| Community onset | 1,879 (46.3%) |
| Health care onset | 2,182 (53.7%) |
| Recent hospitalization (≤90 days)^c^ | 1,534 (37.8%) |
| Most recent prior discharge disposition^d^ |  |
| Nursing home | 308 (20.1%) |
| Long-term care hospital | 28 (1.8%) |
| ICU-acquired^c^ | 861 (21.2%) |
| ICU stay during hospitalization^c^ | 2,045 (50.4%) |
| ICU stay during the 13 days after the index date^c^ | 1,578 (38.9%) |
| COVID-19–related hospitalization^c^ | 271 (6.7%) |
| Recurrence | 1,110 (3.97%) |
| Mortality^c^ |  |
| In-hospital | 1,065 (26.2%) |
| 30-day | 1,225 (30.2%) |
| Elixhauser Comorbidity Index, mean (SD)^e^ | 11.5 (13.62) |
| Comorbidities^e^ |  |
| Anemias | 1832 (47.9%) |
| Diabetes, uncomplicated | 1414 (37.0%) |
| Hypertension, uncomplicated | 1224 (32.0%) |
| Coagulopathy | 1116 (29.2%) |
| Weight loss | 1022 (26.7%) |
| Renal failure, moderate | 741 (19.4%) |
| Solid tumor without metastasis, malignant | 549 (14.4%) |
| Peripheral vascular disease | 501 (13.1%) |
| Chronic pulmonary disease | 20 (0.52%) |
| Heart failure | 365 (9.5%) |
| Liver disease, mild | 130 (3.4%) |
| Valvular disease | 325 (8.5%) |
| Hypothyroidism | 439 (11.5%) |
| Renal failure, severe | 418 (10.9%) |
| Diabetes, complicated | 348 (9.1%) |
| Obesity | 4445 (11.6%) |
| Cerebrovascular disease | 259 (6.8%) |
| Chronic blood loss anemia (iron deficiency) | 271 (7.0%) |
| Metastatic cancer | 153 (4.0%) |
| Peptic ulcer disease | 196 (5.1%) |
| Hypertension, complicated | 181 (4.7%) |
| Pulmonary circulation disease | 153 (4.0%) |
| Depression | 113 (2.9%) |
| Other thyroid disorders | 124 (3.2%) |
| Liver disease, moderate to severe | 158 (4.1%) |
| Other neurological disorders | 98 (2.6%) |
| Autoimmune conditions | 50 (1.3%) |
| Leukemia | 83 (2.1%) |
| Paralysis | 66 (1.7%) |
| Psychosis | 70 (1.8%) |
| Neurological disorders affecting movement | 84 (2.2%) |
| Seizures and epilepsy | 42 (1.1%) |
| Solid organ transplant | 50 (1.3%) |
| Dementia | 36 (0.9%) |
| Lymphoma | 50 (1.3%) |
| Drug abuse | 27 (0.7%) |
| Acquired immune deficiency syndrome | 21 (0.5%) |
| Solid tumor without metastasis, in situ | 17 (0.4%) |
| Alcohol abuse | 9 (0.2%) |

Abbreviation list: ICU = intensive care unit, IQR = interquartile range (25th–75th percentile), SD = standard deviation

^a^ Race in Epic Cosmos is self-reported according to the Centers for Disease Control and Prevention (CDC) categories.^2^

^b^ The Social Vulnerability Index (SVI) values reflect the CDC and Agency for Toxic Substances and Disease Registry (ATSDR) overall SVI percentile, which ranges from 0 to 1. Higher values indicate greater vulnerability.^3^ 62 patients did not have information for this variable. The median and IQR were calculated for 3,759 patients.

^c^ Percentages in these categories are based on the total number of cases (N=4,061). Other percentages are calculated based on the total number of patients (N=3,821) as per the Centers for Disease Control and Prevention (CDC) criteria.^4^

^d^ Percentages for the most recent discharge disposition are based on the total of cases with previous hospitalizations within 90 days of the index date (N=1,534).

^e^ Comorbidities were defined using the Elixhauser Comorbidity Index (ECI) from the International Classification of Diseases-10-Clinical Modification (ICD-10-CM) Refined Software.^5^ The ECI score was calculated for each patient’s first case.

## **Supplementary Table 5. Standardized Mean Differences (SMDs) for Covariate Balance: Full vs Sensitivity Cohort**

| **Variable** | **Absolute SMD** | **Balance categories** |
| --- | --- | --- |
| Age, years, mean (SD) | 0.01 | Good Balance (< 0.1) |
| Age ≥ 18 years | 0.01 | Good Balance (< 0.1) |
| Sex, N (%) |  |  |
| Female | 0.01 | Good Balance (< 0.1) |
| Male | 0.01 | Good Balance (< 0.1) |
| Unknown | 0.03 | Good Balance (< 0.1 |
| Race, N (%) |  |  |
| American Indian/Alaska Native | 0.07 | Good Balance (< 0.1) |
| Asian | 0.05 | Good Balance (< 0.1) |
| Black/African American | 0.03 | Good Balance (< 0.1) |
| Native Hawaiian/Pacific Islander | 0.04 | Good Balance (< 0.1) |
| Other | 0.04 | Good Balance (< 0.1) |
| White | 0.05 | Good Balance (< 0.1) |
| Missing value | 0.12 | Acceptable Balance (0.1 - 0.25) |
| Ethnicity |  |  |
| Not Hispanic/Latino | 0.06 | Good Balance (< 0.1) |
| Hispanic/Latino | 0.03 | Good Balance (< 0.1) |
| Unspecified | 0.07 | Good Balance (< 0.1) |
| Social Vulnerability Index, overall percentile, median (IQR) | 0.03 | Good Balance (< 0.1) |
| Onset |  | Good Balance (< 0.1) |
| Community onset | 0.02 |  |
| Health care onset | 0.02 |  |
| Recent hospitalization (≤90 days) | 0.003 | Good Balance (< 0.1) |
| Most recent prior discharge disposition |  |  |
| Nursing home | 0.01 | Good Balance (< 0.1) |
| Long-term care hospital | 0.06 | Good Balance (< 0.1) |
| ICU-acquired | 0.04 |  |
| ICU stay during hospitalization | 0.03 |  |
| ICU stay during the 13 days after the index date | 0.02 |  |
| COVID-19–related hospitalization | 0.006 | Good Balance (< 0.1) |
| Recurrence | 0.29 | Poor Balance (≥ 0.25) |
| Mortality |  |  |
| In-hospital | 0.001 | Good Balance (< 0.1) |
| 30-day | 0.03 | Good Balance (< 0.1) |
| Elixhauser Comorbidity Index, mean (SD) | 0.11 | Acceptable Balance (0.1 - 0.25) |
| Comorbidities |  |  |
| Anemias | 0.15 | Acceptable Balance (0.1 - 0.25) |
| Diabetes, uncomplicated | 0.03 | Good Balance (< 0.1) |
| Hypertension, uncomplicated | 0.02 | Good Balance (< 0.1) |
| Coagulopathy | 0.04 | Good Balance (< 0.1) |
| Weight loss | 0.005 | Good Balance (< 0.1) |
| Renal failure, moderate | 0.003 | Good Balance (< 0.1) |
| Solid tumor without metastasis, malignant | 0.10 | Acceptable Balance (0.1 - 0.25) |
| Peripheral vascular disease | 0.10 | Acceptable Balance (0.1 - 0.25) |
| Chronic pulmonary disease | 0.53 | Poor Balance (≥ 0.25) |
| Heart failure | 0.12 | Acceptable Balance (0.1 - 0.25) |
| Liver disease, mild | 0.36 | Poor Balance (≥ 0.25) |
| Valvular disease | 0.15 | Acceptable Balance (0.1 - 0.25) |
| Hypothyroidism | 0.01 | Good Balance (< 0.1) |
| Renal failure, severe | 0.001 | Good Balance (< 0.1) |
| Diabetes, complicated | 0.04 | Good Balance (< 0.1) |
| Obesity | 0.05 | Good Balance (< 0.1) |
| Cerebrovascular disease | 0.05 | Good Balance (< 0.1) |
| Chronic blood loss anemia (iron deficiency) | 0.004 | Good Balance (< 0.1) |
| Metastatic cancer | 0.05 | Good Balance (< 0.1) |
| Peptic ulcer disease | 0.006 | Good Balance (< 0.1) |
| Hypertension, complicated | 0.02 | Good Balance (< 0.1) |
| Pulmonary circulation disease | 0.03 | Good Balance (< 0.1) |
| Depression | 0.04 | Good Balance (< 0.1) |
| Other thyroid disorders | 0.02 | Good Balance (< 0.1) |
| Liver disease, moderate to severe | 0.02 | Good Balance (< 0.1) |
| Other neurological disorders | 0.01 | Good Balance (< 0.1) |
| Autoimmune conditions | 0.11 | Acceptable Balance (0.1 - 0.25) |
| Leukemia | 0.002 | Good Balance (< 0.1) |
| Paralysis | 0.03 | Good Balance (< 0.1) |
| Psychosis | 0.01 | Good Balance (< 0.1) |
| Neurological disorders affecting movement | 0.01 | Good Balance (< 0.1) |
| Seizures and epilepsy | 0.03 | Good Balance (< 0.1) |
| Solid organ transplant | 0.01 | Good Balance (< 0.1) |
| Dementia | 0.03 | Good Balance (< 0.1) |
| Lymphoma | 0.01 | Good Balance (< 0.1) |
| Drug abuse | 0.02 | Good Balance (< 0.1) |
| Acquired immune deficiency syndrome | 0.01 | Good Balance (< 0.1) |
| Solid tumor without metastasis, in situ | 0.02 | Good Balance (< 0.1) |
| Alcohol abuse | 0.009 | Good Balance (< 0.1) |

Standardized mean differences (SMDs) were calculated in R using the cobalt package. Balance was categorized by absolute SMD as good (<0.10), acceptable (0.10–<0.25), or poor (≥0.25). Abbreviation list: SMD = Standard mean difference.

## **Supplementary Table 6.** **Standardized Mean Differences (SMDs) for Covariate Balance Year by Year in outcomes**

| **Year** | **Proportion**  **Full cohort** | **Proportion**  **Subset** | **Absolute SMD** | **Balance categories** |
| --- | --- | --- | --- | --- |
| ICU-acquired | | | | |
| 2015 | 0.17 | 0.14 | 0.081 | Good Balance (< 0.1) |
| 2016 | 0.18 | 0.25 | 0.183 | Acceptable Balance (0.1 - 0.25) |
| 2017 | 0.18 | 0.15 | 0.071 | Good Balance (< 0.1) |
| 2018 | 0.21 | 0.19 | 0.069 | Good Balance (< 0.1) |
| 2019 | 0.21 | 0.16 | 0.127 | Acceptable Balance (0.1 - 0.25) |
| 2020 | 0.28 | 0.23 | 0.115 | Acceptable Balance (0.1 - 0.25) |
| 2021 | 0.27 | 0.28 | 0.001 | Good Balance (< 0.1) |
| 2022 | 0.25 | 0.24 | 0.022 | Good Balance (< 0.1) |
| 2023 | 0.22 | 0.19 | 0.079 | Good Balance (< 0.1) |
| 2024 | 0.23 | 0.20 | 0.065 | Good Balance (< 0.1) |
| ICU stay during hospitalization | | | | |
| 2015 | 0.44 | 0.43 | 0.015 | Good Balance (< 0.1) |
| 2016 | 0.43 | 0.52 | 0.189 | Acceptable Balance (0.1 - 0.25) |
| 2017 | 0.46 | 0.47 | 0.030 | Good Balance (< 0.1) |
| 2018 | 0.51 | 0.50 | 0.012 | Good Balance (< 0.1) |
| 2019 | 0.51 | 0.44 | 0.125 | Acceptable Balance (0.1 - 0.25) |
| 2020 | 0.56 | 0.52 | 0.071 | Good Balance (< 0.1) |
| 2021 | 0.55 | 0.53 | 0.039 | Good Balance (< 0.1) |
| 2022 | 0.55 | 0.54 | 0.026 | Good Balance (< 0.1) |
| 2023 | 0.51 | 0.46 | 0.107 | Acceptable Balance (0.1 - 0.25) |
| 2024 | 0.53 | 0.54 | 0.010 | Good Balance (< 0.1) |
| ICU stay during the 13 days after the index date | | | | |
| 2015 | 0.33 | 0.34 | 0.017 | Good Balance (< 0.1) |
| 2016 | 0.32 | 0.37 | 0.094 | Good Balance (< 0.1) |
| 2017 | 0.35 | 0.38 | 0.055 | Good Balance (< 0.1) |
| 2018 | 0.39 | 0.41 | 0.041 | Good Balance (< 0.1) |
| 2019 | 0.39 | 0.35 | 0.075 | Good Balance (< 0.1) |
| 2020 | 0.45 | 0.40 | 0.100 | Acceptable Balance (0.1 - 0.25) |
| 2021 | 0.44 | 0.43 | 0.014 | Good Balance (< 0.1) |
| 2022 | 0.42 | 0.41 | 0.022 | Good Balance (< 0.1) |
| 2023 | 0.40 | 0.35 | 0.102 | Acceptable Balance (0.1 - 0.25) |
| 2024 | 0.41 | 0.39 | 0.038 | Good Balance (< 0.1) |
| In-hospital mortality | | | | |
| 2015 | 0.20 | 0.21 | 0.025 | Good Balance (< 0.1) |
| 2016 | 0.21 | 0.28 | 0.153 | Acceptable Balance (0.1 - 0.25) |
| 2017 | 0.20 | 0.15 | 0.120 | Acceptable Balance (0.1 - 0.25) |
| 2018 | 0.22 | 0.21 | 0.014 | Good Balance (< 0.1) |
| 2019 | 0.22 | 0.21 | 0.025 | Good Balance (< 0.1) |
| 2020 | 0.30 | 0.25 | 0.104 | Acceptable Balance (0.1 - 0.25) |
| 2021 | 0.33 | 0.34 | 0.022 | Good Balance (< 0.1) |
| 2022 | 0.31 | 0.31 | 0.003 | Good Balance (< 0.1) |
| 2023 | 0.25 | 0.23 | 0.046 | Good Balance (< 0.1) |
| 2024 | 0.26 | 0.28 | 0.035 | Good Balance (< 0.1) |
| 2015 | 0.20 | 0.21 | 0.025 | Good Balance (< 0.1) |
| 2016 | 0.21 | 0.28 | 0.153 | Acceptable Balance (0.1 - 0.25) |
| 30-day mortality | | | | |
| 2015 | 0.25 | 0.24 | 0.007 | Good Balance (< 0.1) |
| 2016 | 0.26 | 0.29 | 0.065 | Good Balance (< 0.1) |
| 2017 | 0.25 | 0.20 | 0.119 | Acceptable Balance (0.1 - 0.25) |
| 2018 | 0.27 | 0.24 | 0.059 | Good Balance (< 0.1) |
| 2019 | 0.28 | 0.26 | 0.044 | Good Balance (< 0.1) |
| 2020 | 0.36 | 0.30 | 0.110 | Acceptable Balance (0.1 - 0.25) |
| 2021 | 0.38 | 0.38 | 0.002 | Good Balance (< 0.1) |
| 2022 | 0.34 | 0.34 | 0.009 | Good Balance (< 0.1) |
| 2023 | 0.31 | 0.27 | 0.081 | Good Balance (< 0.1) |
| 2024 | 0.32 | 0.32 | 0.004 | Good Balance (< 0.1) |

Standardized mean differences (SMDs) were calculated in R using the cobalt package. Balance was categorized by absolute SMD as good (<0.10), acceptable (0.10–<0.25), or poor (≥0.25). The proportions represent the rate of occurrence of each variable in their respective cohorts. Abbreviation list: SMD = Standard mean difference.

## **Supplementary Table 7. Temporal Concordance Analysis Between Full Cohort and Subset Populations from 2015-2024**

| **Variable** | **Slope Interaction  p-value** | **Slope interpretation** | **Direction agreement** | **Spearman’s rho (ρ)** | **Spearman interpretation** | **Spearman  p-value** |
| --- | --- | --- | --- | --- | --- | --- |
| Age | 0.0334 | Evidence that slopes differ (non-parallel trends) | Both ↑ | 0.927 | Strong shape concordance | 0.0001 |
| Gender | | | | | | |
| Male | 0.9868 | No evidence of different slopes (consistent with parallel trends) | Both ↑ | 0.396 | Weak shape concordance | 0.2568 |
| Female | 0.9774 | No evidence of different slopes (consistent with parallel trends) | Both ↓ | 0.328 | Weak shape concordance | 0.3544 |
| Race | | | | | | |
| American Indian or Alaska Native | 0.6149 | No evidence of different slopes (consistent with parallel trends) | Both ↓ | 0.357 | Weak shape concordance | 0.4316 |
| Asian | 0.0102 | Evidence that slopes differ (non-parallel trends) | Discordant | 0.164 | Poor shape concordance | 0.6515 |
| Black or African American | 0.8674 | No evidence of different slopes (consistent with parallel trends) | Both ↑ | 0.406 | Moderate shape concordance | 0.2443 |
| Native Hawaiian or Other Pacific Islander | 0.1053 | No evidence of different slopes (consistent with parallel trends) | Discordant | -0.400 | Poor shape concordance | 0.6000 |
| Other Race | 0.2997 | No evidence of different slopes (consistent with parallel trends) | Both ↑ | 0.883 | Strong shape concordance | 0.0016 |
| White | 0.6793 | No evidence of different slopes (consistent with parallel trends) | Both ↓ | 0.486 | Moderate shape concordance | 0.1541 |
| Ethnicity | | | | | | |
| Hispanic or Latino | 0.0939 | No evidence of different slopes (consistent with parallel trends) | Both ↑ | 0.152 | Poor shape concordance | 0.6761 |
| Not Hispanic or Latino | 0.0431 | Evidence that slopes differ (non-parallel trends) | Both ↓ | 0.176 | Poor shape concordance | 0.6272 |
| Onset | | | | | | |
| Community onset | 0.8537 | No evidence of different slopes (consistent with parallel trends) | Both ↓ | 0.418 | Moderate shape concordance | 0.2291 |
| Health care onset | 0.8537 | No evidence of different slopes (consistent with parallel trends) | Both ↑ | 0.418 | Moderate shape concordance | 0.2291 |
| ICU variables | | | | | | |
| ICU-acquired | 0.5849 | No evidence of different slopes (consistent with parallel trends) | Both ↑ | 0.612 | Moderate shape concordance | 0.0600 |
| ICU stay during hospitalization | 0.1530 | No evidence of different slopes (consistent with parallel trends) | Both ↑ | 0.648 | Moderate shape concordance | 0.0425 |
| ICU stay during the 13 days after the index date | 0.2502 | No evidence of different slopes (consistent with parallel trends) | Both ↑ | 0.636 | Moderate shape concordance | 0.0479 |
| Mortality | | | | | | |
| In hospital | 0.7936 | No evidence of different slopes (consistent with parallel trends) | Both ↑ | 0.839 | Strong shape concordance | 0.0024 |
| 30-day | 0.8903 | No evidence of different slopes (consistent with parallel trends) | Both ↑ | 0.842 | Strong shape concordance | 0.0022 |

Slope interaction p-value is the p-value for the calendar year x cohort interaction term from a linear model fit to the annual series. It was interpreted as p < 0.05, evidence that slopes differ (non-parallel trends); and p ≥ 0.05, no evidence of different slopes (consistent with parallel trends). This interaction tests whether the rate of change over time differs between cohorts. Direction agreement was derived from the sign of the cohort-specific year slopes from the same linear model and indicates whether both cohorts are trending in the same direction (both ↑ increasing, both ↓ decreasing, both flat, or discordant). Spearman’s rho (ρ) summarizes monotonic concordance between the two cohorts’ annual series (i.e., whether the series move together in a similar shape over time), and was categorized as ρ ≥ 0.70 strong, 0.40–0.69 moderate, 0.20–0.39 weak, and ρ < 0.20 poor concordance.^6^

**Supplementary Table 8**. **Antifungal resistance of *Candida albicans* isolates, 2015-2024**

| **Year** | **Fluc.** | **Vori.** | **Anidula.** | **Caspo.** | **Mica.** | **MDR** |
| --- | --- | --- | --- | --- | --- | --- |
| 2015 | 4.3%  (14/324) | 5.2% (11/211) | 0%  (0/48) | 0.7%  (1/147) | 1.8%  (1/55) | 0%  (0/185) |
| 2016 | 2.0%  (9/445) | 2.5% (7/277) | 0%  (0/57) | 0%  (0/185) | 0%  (0/75) | 0%  (0/233) |
| 2017 | 3.1% (19/612) | 2.9% (12/416) | 0%  (0/80) | 0%  (0/325) | 0.5%  (1/190) | 0.2%  (1/431) |
| 2018 | 3.7% (25/676) | 3.2% (15/461) | 0%  (0/72) | 0%  (0/353) | 0%  (0/204) | 0%  (0/470) |
| 2019 | 1.2% (10/829) | 1.2% (7/599) | 1.16%  (1/86) | 0.2%  (1/491) | 0.6%  (2/323) | 0%  (0/619) |
| 2020 | 2.1% (21/998) | 1.98% (14/706) | 0%  (0/166) | 0.2%  (1/496) | 0.7%  (4/549) | 0%  (0/773) |
| 2021 | 1.9% (23/1,187) | 2.07% (16/773) | 0%  (0/150) | 0%  (0/520) | 0.1%  (1/732) | 0%  (0/974) |
| 2022 | 1.4%  (19/1,350) | 1.2% (10/865) | 2.7%  (4/150) | 1.2%  (7/569) | 1.1%  (10/883) | 0%  (0/1,138) |
| 2023 | 2.1% (28/1,336) | 1.3% (11/825) | 0.6%  (1/172) | 0.2%  (1/499) | 0.4%  (4/1,045) | 0%  (0/1,169) |
| 2024 | 2.2% (34/1,509) | 1.8% (15/850) | 0.4%  (1/232) | 0.2%  (1/545) | 0.4%  (5/1,252) | 0.15%  (2/1,326) |

Abbreviations: Anidula., anidulafungin; caspo., caspofungin; fluc., fluconazole; MDR, multidrug-resistant (defined by CDC as resistance to fluconazole and any echinocandin^1^); mica., micafungin; vori., voriconazole.

## **Supplementary Table 9.** **Antifungal resistance of *Nakaseomyces glabratus* isolates, 2015-2024**

| **Year** | **Fluc.** | **Anidula.** | **Caspo.** | **Mica.** | **MDR** |
| --- | --- | --- | --- | --- | --- |
| 2015 | 15.6%  (33/211) | 8.4%  (6/71) | 10.8%  (8/74) | 8.3%  (8/96) | 1.4%  (2/141) |
| 2016 | 17.8%  (56/314) | 2.5%  (2/79) | 1.4%  (2/138) | 3.2%  (4/125) | 0.48%  (1/207) |
| 2017 | 13.6%  (59/435) | 3.5%  (3/85) | 2.0%  (5/246) | 5.3%  (10/189) | 0.6%  (2/332) |
| 2018 | 16.4%  (83/506) | 0%  (0/91) | 4.8%  (16/330) | 7.3%  (18/247) | 2.5%  (10/400) |
| 2019 | 11.7%  (82/703) | 1.0%  (1/98) | 4.6%  (19/410) | 5.9%  (22/374) | 1.3%  (7/556) |
| 2020 | 10.7%  (71/664) | 4.0%  (5/124) | 3.7%  (14/378) | 4.1%  (19/463) | 1.5%  (9/585) |
| 2021 | 11.1%  (90/812) | 7.6%  (12/158) | 4.9%  (22/447) | 4.4%  (28/636) | 1.3%  (10/766) |
| 2022 | 10.3%  (93/901) | 5.1%  (7/137) | 5.0%  (19/383) | 4.9%  (38/780) | 0.8%  (7/855) |
| 2023 | 11.4% (114/1,002) | 5.9%  (12/203) | 6.0%  (23/380) | 3.7% (40/1,068) | 1.4%  (13/954) |
| 2024 | 9.6% (113/1,179) | 6.1%  (16/260) | 6.0%  (26/427) | 3.3% (44/1,312) | 1.6% (18/1,148) |

Abbreviations: Anidula., anidulafungin; caspo., caspofungin; fluc., fluconazole; MDR, multidrug-resistant (defined by CDC as resistance to fluconazole and any echinocandin^1^); mica., micafungin; vori., voriconazole

**Supplementary Table 10.** **Antifungal resistance of *Pichia kudriavzevii* isolates, 2015-2024**

| **Year** | **Vori.** | **Anidula.** | **Caspo.** | **Mica.** | **MDR** |
| --- | --- | --- | --- | --- | --- |
| 2015 | 6.7%  (1/15) | 0%  (0/9) | 0%  (0/5) | 0%  (0/11) | 0%  (0/3) |
| 2016 | 5.3%  (1/19) | 0%  (0/4) | 0%  (0/13) | 0%  (0/8) | 0%  (0/7) |
| 2017 | 13.3%  (2/15) | 0%  (0/3) | 0%  (0/16) | 0%  (0/8) | 0%  (0/7) |
| 2018 | 4.3%  (1/23) | 0%  (0/1) | 0%  (0/20) | 0%  (0/9) | 0%  (0/9) |
| 2019 | 3.2%  (1/31) | 0%  (0/4) | 5.0%  (1/20) | 0%  (0/16) | 0%  (0/20) |
| 2020 | 2.9%  (1/34) | 0%  (0/9) | 5.6%  (1/18) | 0%  (0/24) | 0%  (0/13) |
| 2021 | 1.7%  (1/59) | 0%  (0/8) | 7.4%  (2/27) | 0%  (0/56) | 0%  (0/43) |
| 2022 | 9.4%  (5/53) | 0%  (0/10) | 5.0%  (1/20) | 3.6%  (2/56) | 2.6%  (1/39) |
| 2023 | 2.3%  (2/86) | 0%  (0/13) | 9.1%  (3/33) | 0.95%  (1/105) | 2.1%  (1/48) |
| 2024 | 1.2%  (1/81) | 0%  (0/16) | 14.8%  (4/27) | 0%  (0/115) | 0%  (0/64) |

Abbreviations: Anidula., anidulafungin; caspo., caspofungin; fluc., fluconazole; MDR, multidrug-resistant (defined by CDC as resistance to fluconazole and any echinocandin^1^); mica., micafungin; vori., voriconazole.

**Supplementary Table 11.** **Antifungal resistance of *Candida parapsilosis* isolates, 2015-2024**

| Year | Fluc. | Vori. | Anidula. | Caspo. | Mica. | MDR |
| --- | --- | --- | --- | --- | --- | --- |
| 2015 | 2.5%  (3/118) | 1.2%  (1/84) | 0%  (0/15) | 0%  (0/57) | 0%  (0/30) | 0%  (0/81) |
| 2016 | 5.1%  (8/156) | 0.9%  (1/115) | 0%  (0/15) | 0%  (0/75) | 0%  (0/32) | 0%  (0/96) |
| 2017 | 4.2%  (11/260) | 3.3%  (7/213) | 6.0%  (2/33) | 0.6%  (1/155) | 4.3%  (3/70) | 0.5%  (1/191) |
| 2018 | 9.3%  (25/269) | 5.7%  (12/211) | 0%  (0/40) | 0%  (0/161) | 1.0%  (1/98) | 0%  (0/200) |
| 2019 | 6.5%  (21/321) | 3.7%  (9/242) | 0%  (0/37) | 0%  (0/185) | 0%  (0/125) | 0%  (0/241) |
| 2020 | 9.4%  (32/342) | 9.5%  (22/232) | 0%  (0/57) | 0%  (0/160) | 0.5%  (1/191) | 0%  (0/265) |
| 2021 | 13.9%  (68/490) | 11.3%  (41/363) | 0%  (0/65) | 0.5%  (1/212) | 0.3%  (1/367) | 0.2%  (1/425) |
| 2022 | 8.2%  (43/522) | 7.2%  (26/362) | 0%  (0/52) | 0%  (0/198) | 1.5%  (6/392) | 0.4%  (2/440) |
| 2023 | 8.3%  (52/624) | 4.7%  (20/426) | 0%  (0/98) | 0%  (0/244) | 0.9%  (5/530) | 0.4%  (2/562) |
| 2024 | 10.8%  (62/574) | 6.2%  (22/353) | 0%  (0/86) | 0%  (0/195) | 1.3%  (7/540) | 0.4%  (2/520) |

Abbreviations: Anidula., anidulafungin; caspo., caspofungin; fluc., fluconazole; MDR, multidrug-resistant (defined by CDC as resistance to fluconazole and any echinocandin^1^); mica., micafungin; vori., voriconazole.

**Supplementary Table 12**. **Antifungal resistance of *Candida tropicalis* isolates, 2015-2024**

| **Year** | **Fluc.** | **Vori.** | **Anidula.** | **Caspo.** | **Mica.** | **MDR** |
| --- | --- | --- | --- | --- | --- | --- |
| 2015 | 6.2%  (4/64) | 6.2%  (3/48) | 0%  (0/4) | 0%  (0/24) | 0%  (0/11) | 0%  (0/32) |
| 2016 | 4.8%  (4/83) | 1.8%  (1/54) | 16.7%  (1/6) | 8.3%  (2/24) | 6.7%  (1/15) | 0%  (0/36) |
| 2017 | 10.5%  (15/143) | 13.9%  (16/115) | 0%  (0/22) | 0%  (0/79) | 0%  (0/51) | 0%  (0/108) |
| 2018 | 13.6%  (18/132) | 18.5%  (17/92) | 0%  (0/12) | 0%  (0/67) | 1.96%  (1/51) | 1.1%  (1/93) |
| 2019 | 15.9%  (24/151) | 16.5%  (16/97) | 0%  (0/17) | 0%  (0/76) | 0%  (0/62) | 0%  (0/99) |
| 2020 | 13.7%  (24/175) | 15.5%  (20/129) | 0%  (0/19) | 0%  (0/83) | 0%  (0/111) | 0%  (0/148) |
| 2021 | 11.4%  (25/220) | 14.0%  (20/143) | 0%  (0/25) | 0%  (0/82) | 0.7%  (1/138) | 0%  (0/183) |
| 2022 | 10.5%  (25/237) | 13.6%  (21/154) | 3.4%  (1/29) | 4.0%  (3/74) | 1.7%  (3/178) | 0.5%  (1/212) |
| 2023 | 14.0%  (39/279) | 11.9%  (23/193) | 2.2%  (1/45) | 0.9%  (1/113) | 0.8%  (2/257) | 0%  (0/260) |
| 2024 | 18.1%  (58/321) | 22.6%  (43/190) | 0%  (0/53) | 1.2%  (1/82) | 1.0%  (3/289) | 0.7%  (2/285) |

Abbreviations: Anidula., anidulafungin; caspo., caspofungin; fluc., fluconazole; MDR, multidrug-resistant (defined by CDC as resistance to fluconazole and any echinocandin^1^); mica., micafungin; vori., voriconazole.

## **Supplementary Table 13. Antifungal resistance of *Candida auris* isolates, 2022-2024**

| **Year** | **Fluc.** | **Vori.** | **Anidula.** | **Caspo.** | **Mica.** | **MDR** |
| --- | --- | --- | --- | --- | --- | --- |
| 2022 | 62.5%  (5/8) | 0%  (0/2) | 0%  (0/5) | 20%  (1/3) | 0%  (0/9) | 12.5%  (1/8) |
| 2023 | 75.8% (25/33) | 25%  (1/4) | 7.7%  (1/13) | 8.3%  (1/9) | 11.5%  (3/26) | 11.5%  (3/26) |
| 2024 | 100%  (58/58) | 0%  (0/1) | 0%  (0/29) | 0%  (0/29) | 9.1%  (4/44) | 9.1%  (4/44) |

Abbreviations: Anidula., anidulafungin; caspo., caspofungin; fluc., fluconazole; MDR, multidrug-resistant (defined by CDC as resistance to fluconazole and any echinocandin^1^); mica., micafungin; vori., voriconazole.

## **Supplementary Table 14.** **The total number of *Candida* species isolates with missing or invalid antifungal susceptibility results that were excluded from the analysis**

| ***Candida* species** | **Number of isolates in the original dataset** | **Number of excluded isolates  N (%)** | **Total isolates in the antifungal susceptibility analysis** |
| --- | --- | --- | --- |
| *Candida albicans* | 12,798 | 3,457 (27) | 9,341 |
| *Nakaseomyces glabratus* | 10,147 | 2,465 (24.3) | 7,682 |
| *Candida parapsilosis* | 4,745 | 1,000 (21.1) | 3,745 |
| *Candida tropicalis* | 2,414 | 542 (22.5) | 1,872 |
| *Candida auris* | 639 | 538 (84.2) | 101 |
| *Pichia kudriavzevii* | 666 | 119 (17.9) | 547 |

# **REFERENCES**

1. Centers for Disease Control and Prevention, National Center for Health Statistics. Geographic division or region. Health, United States. [Internet]. Atlanta (GA): CDC; [cited 2025 Sep 15]. Available from: https://www.cdc.gov/nchs/hus/sources-definitions/geographic-region.htm
2. Centers for Disease Control and Prevention. Race and Ethnicity Code Set Version 1.0: Background and Purpose. [Internet]. Atlanta (GA): CDC; 2000 Mar [cited 2025 Sep 15]. Available from: https://www.cdc.gov/phin/media/pdfs/2024/09/CDC-Race-Ethnicity-Background-and-Purpose_Pgs-1-to-3.pdf
3. Centers for Disease Control and Prevention; Agency for Toxic Substances and Disease Registry. CDC/ATSDR Social Vulnerability Index (SVI) 2020 documentation. [Internet]. Atlanta (GA): CDC/ATSDR; 2022 [cited 2025 Sep 15]. Available from: https://svi.cdc.gov/map25/data/docs/SVI2020Documentation_08.05.22.pdf
4. Jenkins EN, Gold JAW, Benedict K, et al. Population-Based Active Surveillance for Culture-Confirmed Candidemia — 10 Sites, United States, 2017–2021. MMWR Surveillance Summaries 2025;74(4):1–15.
5. Agency for Healthcare Research and Quality. Elixhauser Comorbidity Software Refined for ICD-10-CM. Healthcare Cost and Utilization Project (HCUP). [Internet]. Rockville (MD): AHRQ; 2021 [cited 2025 Sep 16]. Available from: https://hcup-us.ahrq.gov/toolssoftware/comorbidityicd10/comorbidity_icd10.jsp
6. Akoglu H. User's guide to correlation coefficients. Turk J Emerg Med. 2018;18(3):91-93.
7. Epic Systems Corporation. Epic Cosmos. [Internet]. Verona (WI): Epic Systems Corporation; [cited 2025 Sep 15]. Available from: <https://cosmos.epic.com/>
8. R Core Team. R for Windows (version 4.4.1). [Internet]. Vienna (AT): R Foundation for Statistical Computing; [cited 2025 Sep 15]. Available from: <https://cran.r-project.org/bin/windows/base/old/4.4.1/>
9. Moin EE, Seewald NJ, Halpern SD. Use of life support and outcomes among patients admitted to intensive care units. JAMA. 2025;333(20):1793-1803.
10. ICD10Data.com. 2025 ICD-10-PCS procedure codes. [Internet]. [place unknown]: ICD10Data.com; 2025 [cited 2025 Sep 16]. Available from: <https://www.icd10data.com/ICD10PCS/Codes>
11. Centers for Disease Control and Prevention. Antifungal susceptibility testing for Candida auris. Candida auris (C. auris). [Internet]. Atlanta (GA): CDC; 2024 [cited 2025 Sep 15]. Available from: <https://www.cdc.gov/candida-auris/hcp/laboratories/antifungal-susceptibility-testing.html>
12. Procop GW, Dufresne P, Berkow EL, et al. Performance standards for antifungal susceptibility testing of yeasts. 3rd ed. CLSI document M27M44S. Wayne (PA): Clinical and Laboratory Standards Institute; 2022.
13. Austin PC. Balance diagnostics for comparing the distribution of baseline covariates between treatment groups in propensity-score matched samples. Stat Med. 2009;28(25):3083-3107.
14. Stuart EA. Matching methods for causal inference: a review and a look forward. Stat Sci. 2010;25(1):1-21.
15. Habibzadeh F. Data distribution: normal or abnormal? J Korean Med Sci. 2024;39(3):e35.
